# A dyad of human-specific *NBPF14* and *NOTCH2NLB* orchestrates cortical progenitor abundance crucial for human neocortex expansion

**DOI:** 10.1126/sciadv.ads7543

**Published:** 2025-03-26

**Authors:** Nesil Eşiyok, Neringa Liutikaite, Christiane Haffner, Jula Peters, Sabrina Heide, Christina Eugster Oegema, Wieland B. Huttner, Michael Heide

**Affiliations:** ^1^ German Primate Center, Leibniz Institute for Primate Research, Kellnerweg 4, D-37077 Göttingen, Germany.; ^2^ Max Planck Institute of Molecular Cell Biology and Genetics, Pfotenhauerstrasse 108, D-01307 Dresden, Germany.

## Abstract

We determined the roles of two coevolved and coexpressed human-specific genes, *NBPF14* and *NOTCH2NLB*, on the abundance of the cortical progenitors that underlie the evolutionary expansion of the neocortex, the seat of higher cognitive abilities in humans. Using automated microinjection into apical progenitors (APs) of embryonic mouse neocortex and electroporation of APs in chimpanzee cerebral organoids, we show that *NBPF14* promotes the delamination of AP progeny, by promoting oblique cleavage plane orientation during AP division, leading to increased abundance of the key basal progenitor type, basal radial glia. In contrast, *NOTCH2NLB* promotes AP proliferation, leading to expansion of the AP pool. When expressed together, *NBPF14* and *NOTCH2NLB* exert coordinated effects, resulting in expansion of basal progenitors while maintaining self-renewal of APs. Hence, these two human-specific genes orchestrate the behavior of APs, and the lineages of their progeny, in a manner essential for the evolutionary expansion of the human neocortex.

## Teaser

Two human-specific genes act together to multiply neural progenitor cells for human brain expansion.

## INTRODUCTION

The remarkable expansion of the cerebral cortex over the past ≈2 millions of years of human evolution culminated in the strongly folded human neocortex, which is three times larger than that of our closest living relative, the chimpanzee (*1*–*5*). This tremendous increase in the size of the human neocortex is thought to be a basis for our higher cognitive abilities (*1*, *6*–*10*). A key process underlying the evolutionary expansion of the neocortex is cortical neurogenesis, which typically occurs during fetal development and involves cortical neural stem and progenitor cells (cNPCs) (*5*–*8*, *11*).

Two main classes of cNPCs can be distinguished within the two principal germinal zones of fetal neocortex: apical progenitors (APs) and basal progenitors (BPs) (*6*–*8*, *12*, *13*). APs reside in the primary germinal zone, the ventricular zone (VZ) lining the ventricle, and comprise the primary cNPCs, the neuroepithelial cells (NECs) (*11*, *14*). With the onset of cortical neurogenesis, NECs transform into apical radial glia (aRG; also called ventricular radial glia) (*6*–*8*, *13*, *15*–*17*). BPs reside in the secondary germinal zone, the subventricular zone (SVZ), which lies basally adjacent to the VZ (*18*, *19*). Two types of BPs can be distinguished: basal radial glia (bRG; also called outer radial glia) and basal intermediate progenitors (bIPs) (*7*, *20*–*22*). In species with an expanded neocortex, notably primates and in particular human, the SVZ becomes subdivided into an inner (iSVZ) and an outer SVZ (oSVZ) (*21*, *23*–*25*). The iSVZ largely corresponds to the SVZ of small brain-containing rodents, and the oSVZ with its abundance of bRG has been implicated in neocortex expansion (*6*, *8*, *21*, *22*, *24*, *26*–*29*).

When considering the role of the various types of cNPCs in the evolutionary expansion of the neocortex and the associated increase in cortical neurogenesis, both the lineage of cNPCs and their modes of division are key (*12*, *30*–*32*). The canonical lineage of cortical neurogenesis is: APs make BPs make neurons. Initially, both NECs and a portion of aRG undergo symmetric proliferative divisions, which eventually increases the number of radial units (*33*, *34*). With the progression of cortical neurogenesis, an increasing proportion of aRG switch to asymmetric self-renewing divisions, which yield one aRG daughter and one newborn BP daughter, with the latter migrating to the SVZ.

Both types of BPs can self-amplify by symmetric proliferative divisions, and can undergo neuron-generating divisions. bRG typically do the latter by asymmetric self-renewing divisions, which yield one bRG daughter and one newborn neuron that then migrates to the cortical plate. bIPs typically generate neurons by symmetric consumptive division (*11*, *12*, *15*, *35*–*37*). In these cNPC lineage and division mode scenarios, it is essential to realize that a mere expansion of the AP pool size, without a concomitant increase in BP generation, is insufficient for neocortex expansion (*6*, *8*, *38*).

In light of the fact that neocortex expansion is particularly relevant in the case of human evolution, a research focus has been to study genomic alterations that have occurred specifically during human evolution and that differentially affect the abundance, behavior, and activity of human cNPCs in comparison to cNPCs of other primates (*19*, *39*, *40*). Such human-specific genomic alterations include both small changes such as nucleotide substitutions, as well as large changes that led to the emergence of novel genes that evolved specifically in the human lineage (*41*–*44*). With regard to the evolutionary expansion of the human neocortex, the latter genes, referred to as human-specific genes, are of particular interest if they are preferentially expressed in human cNPCs as opposed to neurons. While a number of such human-specific genes have been identified over the past decade (*45*) [summarized in Heide and Huttner (*46*)], functional studies dissecting how these genes affect cNPCs have addressed only two of them, *ARHGAP11B* and the *NOTCH2NL* genes (*NOTCH2NLA-C*) (*45*, *47*, *48*). Thus, *ARHGAP11B* has been shown to increase both, the generation of BPs from aRG and the self-renewal of BPs. Hence, the human-specific gene *ARHGAP11B* fulfills the abovementioned criterion that an increase in BPs is essential for neocortex expansion. In support of this concept, *ARHGAP11B* expression in fetuses of the common marmoset increases the size of the neocortex and induces its folding, phenotypes that were found to be associated with a specific increase in the abundance of BPs, notably bRG and in particular in the oSVZ (*44*).

In contrast, in the case of the human-specific *NOTCH2NL* genes, it has been shown that expression of the *NOTCH2NLA* variant in the embryonic mouse neocortex increases the proportion of cycling BPs in the SVZ, but not of APs in the VZ (*45*). Conversely, two comprehensive studies (*47*, *48*) have reported that the expression of the *NOTCH2NLB* variant in embryonic mouse neocortex increases aRG abundance in the VZ without increasing BP abundance in the SVZ. Mechanistically, NOTCH2NLB has been shown to activate Notch signaling by inhibiting the interaction between Notch and Dll1 in a cell-autonomous manner, thereby promoting the self-renewal of aRG (*47*, *48*). In light of the notion that a mere expansion of the AP pool size, without a concomitant increase in BP generation, is insufficient for neocortex expansion, this finding by Suzuki *et al.*, if confirmed, would imply that *NOTCH2NLB* alone is unlikely to be able to cause an expansion of the neocortex during human evolution.

The *NOTCH2NLB* gene, located on the long arm of chromosome 1 at position 1q21.1, lies adjacent to another human-specific gene preferentially expressed in cNPCs, *NBPF14* (*41*, *49*), which shows strongest expression in aRG (*45*). *NBPF14* belongs to the *neuroblastoma breakpoint family* (*NBPF*) of genes. *NBPF* genes are defined by the presence of a protein domain called Olduvai domain (previously known as DUF1220) (*50*), whose molecular function is currently unknown. While the molecular mechanism underlying the function of *NBPF14* has not yet been elucidated, one member of the *NBPF* gene family, *NBPF1*, has been suggested to play a role in metabolic regulation by fine-tuning mitochondrial function, indicating a potential role of Olduvai domain(s) in this context (*51*). Genomic and phylogenetic analyses revealed that a large number of *NBPF* gene family members are found in the primate lineage, with the greatest number of members present in human (*50**,* *52*). It has been suggested that *NBPF14* and *NOTCH2NLB* evolved jointly as a two-gene module in the human lineage (*49*). In light of the coexpression of these two genes in human cNPCs, notably in aRG (*45*, *46*, *49*), the question arises whether these human-specific genes can affect cNPCs in a coordinated, potentially synergistic, manner.

Here, we analyzed the role of *NBPF14* during prenatal neocortical development, focusing on the effects of this human-specific gene on cNPCs and its possible functional interaction with *NOTCH2NLB*. To this end, we have used automated microinjection into aRG of embryonic mouse neocortex and electroporation of chimpanzee cerebral organoids. We confirm that *NOTCH2NLB* amplifies aRG, without increasing the generation of BPs, implying that this human-specific gene alone is unlikely to be able to cause an expansion of the neocortex during human evolution. Our functional analyses reveal that *NBPF14* induces delamination of cNPCs from the VZ by modifying the cleavage plane orientation of mitotic aRG, thereby increasing the abundance of bRG in the developing neocortex. We further provide evidence that the combined expression of *NBPF14* and *NOTCH2NLB* expands the BP pool while maintaining the self-renewal capacity of APs. Our data therefore provide a paradigmatic example of how two coevolved human-specific genes, *NBPF14* and *NOTCH2NLB*, located adjacent to each other on chromosome 1, act in a coordinated and synergistic manner in aRG during cortical development. This concerted action of these genes likely has contributed to the increase in neocortex size during human evolution.

## RESULTS

### *NBPF14*, in contrast to *NOTCH2NLB*, does not promote proliferation of APs

A previous expression analysis of different *NBPF14* isoforms in cNPCs had shown that the *NBPF14* isoform NBPF14-204 (ENST00000616120.4) exhibits the strongest expression of all *NBPF14* isoforms in aRG, bRG, and neurons combined, with the highest expression in aRG (*45*). We therefore focused our analysis on the isoform NBPF14-204 (fig. S1A; for simplicity, hereafter referred to as *NBPF14*) as a candidate gene to regulate the activity and behavior of cNPCs, notably of aRG. To explore the potential role of *NBPF14* and its possible functional interaction with *NOTCH2NLB* on cNPC activity and behavior, we made use of two different experimental approaches, each in a distinct mammalian system that endogenously lacks these two human-specific genes. Thus, we performed (i) automated microinjection of mRNAs into single APs of embryonic mouse neocortex at embryonic day E14.5, and (ii) electroporation of expression plasmids into chimpanzee cerebral organoids at 42 days of culture (fig. S1B).

Using the Autoinjector, a robotic platform for microinjection of single cells in brain tissue (*53*), we microinjected the following mRNAs into single APs in embryonic mouse neocortex: *RFP* mRNA only (control), *RFP* plus *NOTCH2NLB* mRNAs (NOTCH2NLB), *RFP* plus *NBPF14* mRNAs (NBPF14), or *RFP* plus *NOTCH2NLB* plus *NBPF14* mRNAs (NOTCH2NLB + NBPF14). Following automated microinjection, the progeny of the transfected APs (hereafter referred to as control-progeny, NOTCH2NLB-progeny, NBPF14-progeny, or NOTCH2NLB + NBPF14-progeny, respectively) was analyzed after 24 and 48 hours to investigate the effects on mouse APs after one and two cell divisions, respectively (fig. S1B).

For chimpanzee cerebral organoid electroporation, we administered the following expression plasmids into large ventricle-like structures of the organoids: enhanced green fluorescent protein (EGFP) expression plasmid plus either control plasmid (control), *NOTCH2NLB* expression plasmid only (NOTCH2NLB), *NBPF14* expression plasmid only (NBPF14), or *NOTCH2NLB* plus *NBPF14* expression plasmids (NOTCH2NLB + NBPF14), and subsequently performed electroporation to transfect APs in the VZ. After electroporation, organoids were analyzed 2 and 8 days postelectroporation (dpe), depending on the target cell population to be analyzed for potential effects of *NOTCH2NLB* and/or *NBPF14* (fig. S1B).

To confirm that *NOTCH2NLB* and *NBPF14* are expressed following microinjection into single APs of the embryonic mouse neocortex and after electroporation into chimpanzee cerebral organoids, we performed immunofluorescence for red fluorescent protein (RFP; in the case of microinjection) or EGFP (in the case of electroporation), in combination with anti-NOTCH2NL or anti-NBPF antibodies for both experimental systems. We detected expression of both genes in microinjected APs of the embryonic mouse neocortex and in electroporated cells of chimpanzee cerebral organoids (fig. S1, C and D), confirming the functionality of our experimental setups.

**Fig. 1.**
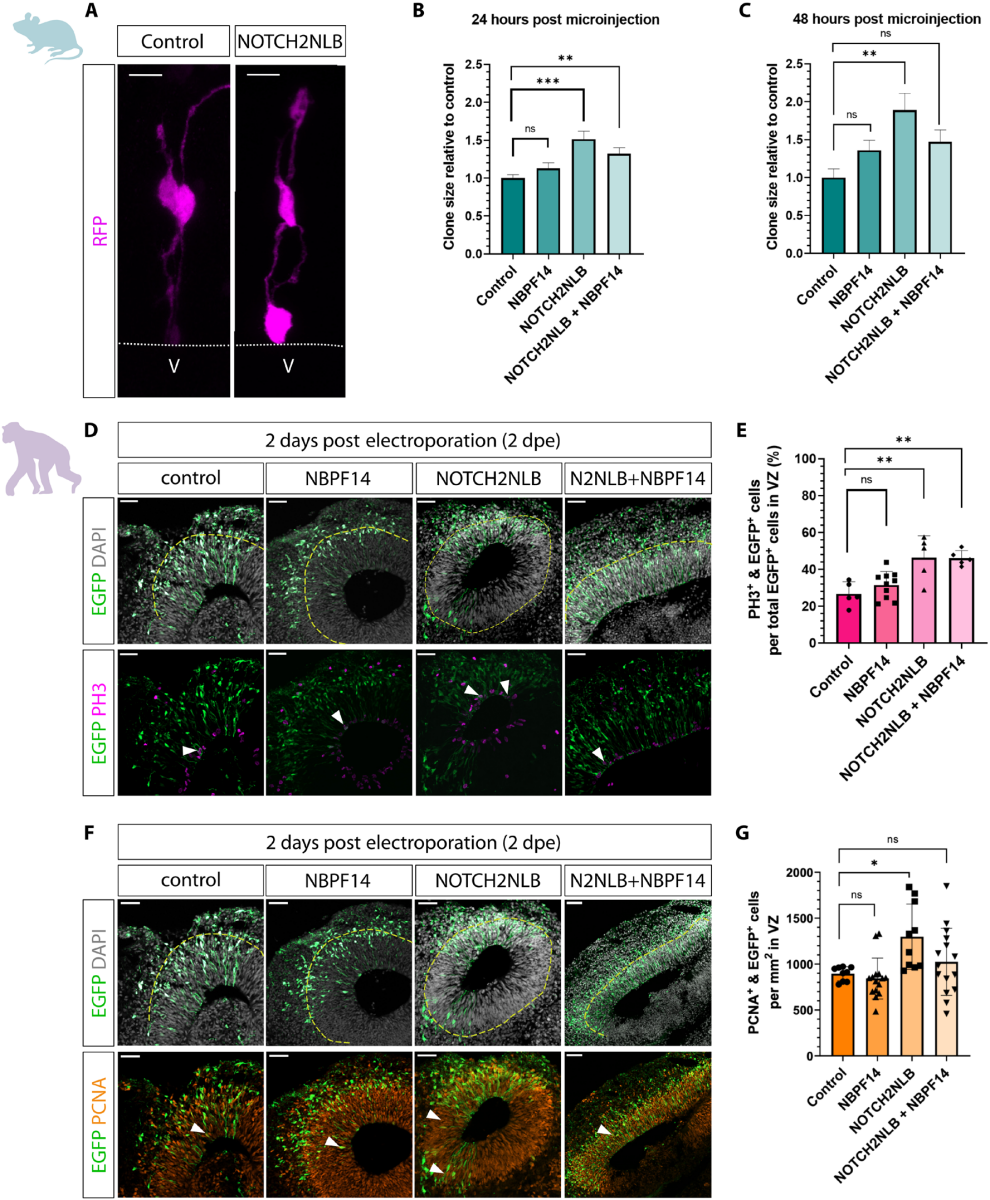
Unlike *NOTCH2NLB*, *NBPF14* does not increase the proliferation of APs in embryonic mouse neocortex and chimpanzee cerebral organoids. (**A**) RFP immunofluorescence 24 hours after automated microinjection (aMI) of *RFP* (control) or *RFP* plus *NOTCH2NLB* mRNAs (NOTCH2NLB) into a single AP of E14.5 mouse neocortex (mNCX). Dashed line, ventricular surface; V, ventricle. Scale bars, 10 μm. (**B** and **C**) Clone size of RFP^+^ cells relative to control, 24 (B) and 48 hours (C) after aMI of E14.5 mNCX of *RFP* (control) or *RFP* plus either *NBPF14*, *NOTCH2NLB*, or *NOTCH2NLB* plus *NBPF14* mRNAs. Data are the mean of 79 control, 41 NBPF14, 67 NOTCH2NLB, and 58 NOTCH2NLB + NBPF14 (B), and 63 control, 75 NBPF14, 43 NOTCH2NLB, and 71 NOTCH2NLB + NBPF14 (C) microinjected APs; error bars, SEM; ns not significant, **P < 0.01 and ***P < 0.001 (Kruskal-Wallis test). Absolute clone size of control was 1.34 (B) and 2.16 (C). (**D** and **F**) Double immunofluorescence for EGFP (green) and either PH3 [magenta, (D)] or PCNA [orange, (F)], combined with DAPI (gray), of 44-day-old chimpanzee cerebral organoids (COs) 2 days after electroporation (dpe) of EGFP plus either control, *NBPF14*, *NOTCH2NLB*, or *NOTCH2NLB* plus *NBPF14* (N2NLB + NBPF14) expression plasmids. Dashed lines, basal boundary of VZ; arrowheads, examples of EGFP and either PH3 (D) or PCNA (F) double-positive cells. Scale bars, 50 μm. (**E**) Percentage of EGFP^+^/PH3^+^ cells within the VZ area of 44-day-old chimpanzee COs 2 dpe. Data are the mean of five control, 10 NBPF14, five NOTCH2NLB, and five NOTCH2NLB + NBPF14 electroporated organoids; error bars, ±SD; ns not significant, **P < 0.01 (ANOVA with Dunnett’s correction). (**G**) EGFP^+^/PCNA^+^ cells within 1-mm^2^ VZ area of 44-day-old chimpanzee COs 2 dpe. Data are the mean of nine control, 15 NBPF14, 10 NOTCH2NLB, and 15 NOTCH2NLB + NBPF14 electroporated organoids; error bars, ±SD; ns not significant, *P < 0.05 (Kruskal-Wallis test).

We next sought to confirm the expression of *NBPF14* in cNPCs of fetal human neocortex at the protein level. Using an anti-NBPF antibody, which detects several members of the NBPF family including NBPF14, we analyzed the localization of NBPFs in the germinal zones of fetal human neocortex. We detected a strong punctate signal in both the VZ and SVZ, i.e., in APs and BPs, respectively, with the immunoreactive puncta in APs being concentrated toward the ventricular surface (fig. S2). These data confirm the mRNA expression pattern (*45*). Given the robust expression pattern of *NBPF14* in aRG (*49*), we asked if *NBPF14*, similar to its genomic neighbor *NOTCH2NLB*, would enhance the proliferation of cNPCs. Accordingly, we first analyzed RFP^+^ cells to determine the number of the progeny derived from each single microinjected AP in embryonic mouse neocortex (Fig. 1A). We found that the NOTCH2NLB-progeny comprised a significantly larger clone size in comparison to control 24 hours after microinjection (Fig. 1B), a difference that became even more pronounced 48 hours after microinjection (Fig. 1C). This finding is in line with previously published data, which suggested that *NOTCH2NLB* plays a role in amplifying APs (*47*, *48*). In contrast to the NOTCH2NLB-progeny, the clone size of the NBPF14-progeny remained similar to control and did not significantly change at either time point analyzed (Fig. 1, B and C). In other words, *NBPF14* does not increase the clone size of the progeny of microinjected APs in embryonic mouse neocortex.

Given the coevolution and coexpression of *NOTCH2NLB* and *NBPF14*, we next asked whether *NBPF14* could perhaps promote the role of *NOTCH2NLB* in increasing the clone size of the progeny of microinjected APs, although it does not increase clone size on its own. We found that the coexpression of *NBPF14* and *NOTCH2NLB* also resulted in a larger clone size in comparison to control 24 hours after microinjection (Fig. 1B), which however was not larger than that observed upon expression of *NOTCH2NLB* alone. This indicates that *NBPF14* does not promote the function of *NOTCH2NLB* in expanding APs in embryonic mouse neocortex. At 48 hours after microinjection, the clone size of the NOTCH2NLB + NBPF14-progeny was, with regard to statistical significance, neither larger in comparison to control nor smaller in comparison to *NOTCH2NLB* expression alone (Fig. 1C and figure legend). In summary, the microinjection data in embryonic mouse neocortex show that *NBPF14*, in contrast to *NOTCH2NLB*, does not increase the clone size of the AP progeny.

Recently, our laboratories demonstrated the usefulness of the electroporation of chimpanzee cerebral organoids to investigate the effects of human-specific genes on cNPC activity and behavior (*54*–*56*). We therefore sought to apply this technique to corroborate the results obtained in embryonic mouse neocortex in a model system that is evolutionarily closer to human. To this end, we generated chimpanzee cerebral organoids and electroporated them with the expression plasmids of *NBPF14* and/or *NOTCH2NLB* as described above. We analyzed chimpanzee cerebral organoids 2 dpe; during this time period, electroporated APs in chimpanzee cerebral organoids undergo one cell cycle to generate EGFP^+^ daughter cells that are almost exclusively located in the VZ (*55*).

We first analyzed changes in the number of mitotic APs using immunofluorescence for the mitosis marker phospho-histone 3 (PH3) (Fig. 1D) and quantified the percentage of EGFP-positive APs at the ventricular surface that were also positive for PH3. Upon expression of *NOTCH2NLB*, we found a significant increase in the percentage of the EGFP-positive NOTCH2NLB-progeny that were PH3 positive, and hence mitotic APs (Fig. 1E). In contrast, the expression of *NBPF14* did not lead to a significant increase in this percentage (Fig. 1E). These results therefore confirmed the findings obtained in the microinjection experiments with embryonic mouse neocortex indicating that *NBPF14* does not increase AP proliferation. However, in contrast to the data in embryonic mouse neocortex, the combined expression of *NOTCH2NLB* and *NBPF14* in chimpanzee cerebral organoids resulted in a significant increase in mitotic APs, similar to the *NOTCH2NLB* condition (Fig. 1E).

To address if the increase in mitotic APs upon expression of *NOTCH2NLB* or *NOTCH2NLB* + *NBPF14* gives rise to an increased number of cycling APs, we next analyzed the electroporated chimpanzee cerebral organoids by immunofluorescence for the proliferation marker proliferating cell nuclear antigen (PCNA; Fig. 1F). Specifically, we quantified the number of PCNA-positive cells within the EGFP^+^ progeny of the electroporated APs in 1 mm^2^ of the electroporated VZ area. Upon expression of *NOTCH2NLB*, we found a significant increase in the number of PCNA^+^ APs in the VZ compared to control-electroporated organoids (Fig. 1G). This finding is further evidence in support of previous studies concluding that *NOTCH2NLB* promotes expansion of APs during human neocortical development (*47*, *48*).

Consistent with our findings in embryonic mouse neocortex (Fig. 1, B and C), expression of *NBPF14* in chimpanzee cerebral organoids did not lead to a significant increase in the number of cycling APs in the VZ (Fig. 1G). Hence, *NBPF14*, unlike its genomic neighbor, *NOTCH2NLB*, does not increase the proliferation of APs in embryonic mouse neocortex and chimpanzee cerebral organoids. In contrast to the expression of *NOTCH2NLB* alone, coexpression of *NOTCH2NLB* plus *NBPF14* also did not result in a significant increase in the number of cycling APs in the VZ of chimpanzee cerebral organoids (Fig. 1G). As the coexpression of *NOTCH2NLB* and *NBPF14* increased the percentage of APs in mitosis (Fig. 1E), this raised the possibility that the combined expression of these two human-specific genes in chimpanzee cerebral organoids increases the generation of non-AP progeny from the mitotic APs, notably BPs, that then leave the VZ. As *NOTCH2NLB* increases the AP pool size (Fig. 1G), we therefore asked whether *NBPF14* increases the delamination of cNPCs from the ventricular surface (*12*, *20*, *21*, *57*).

In the following three sections, we will first describe the effect of *NBPF14* on cNPC delamination and elaborate on our experimental approaches to dissect this process. Applying the same experimental approaches, we will then summarize the lack of effect of *NOTCH2NLB* on cNPC delamination and the effect of *NBPF14* on cNPC delamination in the presence of *NOTCH2NLB*.

### *NBPF14* leads to increased delamination of APs in embryonic mouse neocortex and chimpanzee cerebral organoids

**Fig. 2.**
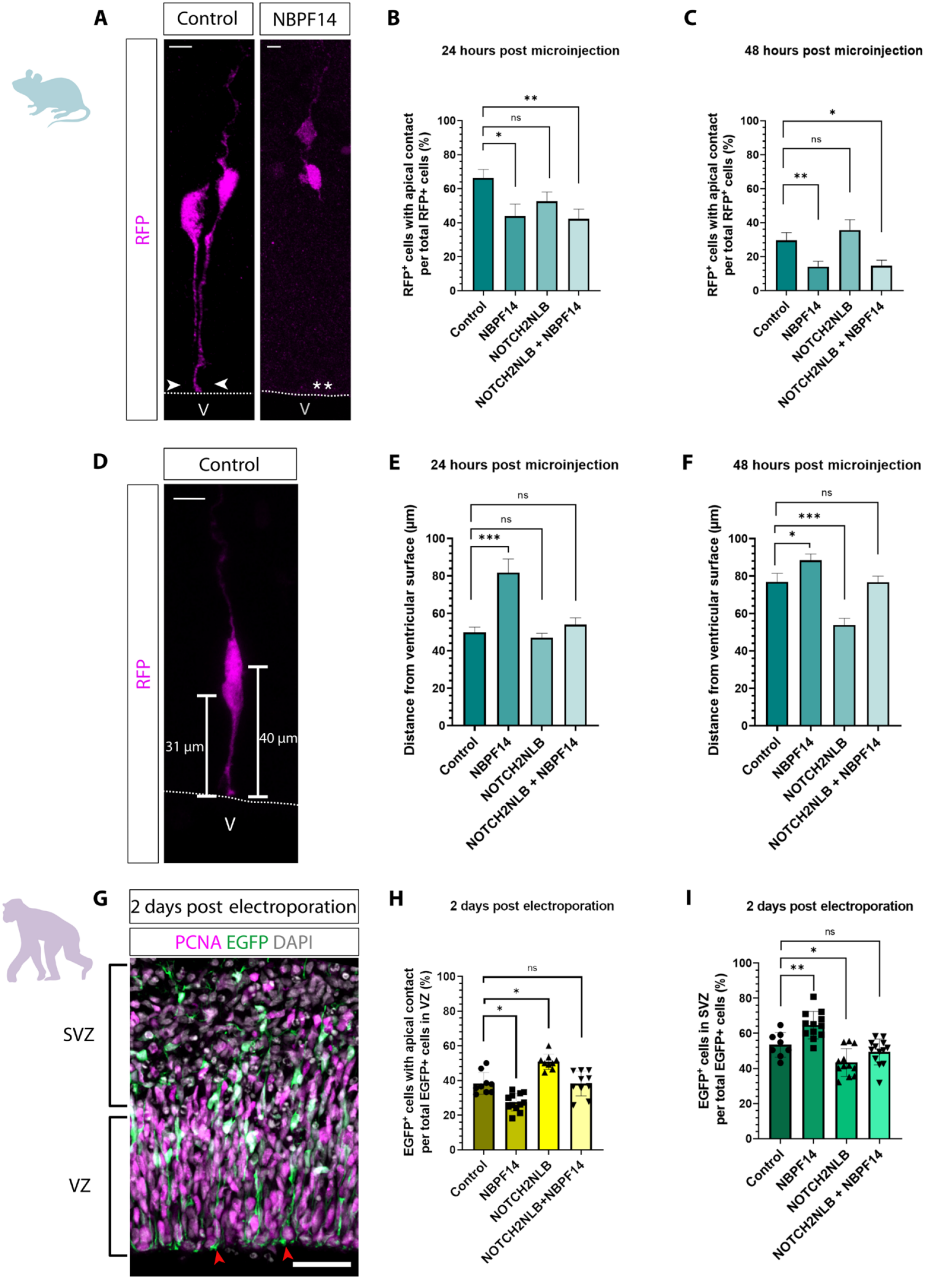
*NBPF14* promotes delamination of AP progeny in embryonic mouse neocortex and chimpanzee cerebral organoids. (**A** and **D**) RFP immunofluorescence 24 hours after automated microinjection (aMI) of *RFP* (control) or *RFP* plus *NBPF14* mRNAs (A), or *RFP* mRNA (D) into APs of E14.5 mouse neocortex (mNCX). Arrowheads, apical contact; dashed lines, ventricular surface (VS); V, ventricle; asterisks, lack of apical contact; bars, distance of cell body center from VS. Scale bars, 10 μm. (**B** and **C**) Percentage of RFP^+^ cells with apical contact 24 (B) and 48 hours (C) after aMI of E14.5 mNCX of *RFP* (control) or *RFP* plus either *NBPF14*, *NOTCH2NLB*, or *NOTCH2NLB* plus *NBPF14* mRNAs. Data, mean of 79/63 control-, 41/75 NBPF14-, 67/43 NOTCH2NLB-, and 58/71 NOTCH2NLB + NBPF14–microinjected APs for (B)/(C), respectively; error bars, SEM; ns not significant, *P < 0.05 and **P < 0.01 (Kruskal-Wallis test). (**E** and **F**) Distance of cell body center of RFP^+^ cells from VS 24 (E) and 48 hours (F) after aMI of E14.5 mNCX using the same mRNAs as in (B) and (C). Data, mean of 106/136 control, 62/205 NBPF14, 136/137 NOTCH2NLB, and 103/192 NOTCH2NLB + NBPF14 AP progeny cells for (E)/(F), respectively; error bars, SEM; ns not significant, *P < 0.05 and ***P < 0.001 (Kruskal-Wallis test). (**G**) EGFP (green) and PCNA (magenta) immunofluorescence, combined with DAPI (gray), of a 44-day-old chimpanzee cerebral organoid (cCO) 2 dpe of EGFP plus control expression plasmids. Brackets, VZ and SVZ; arrowheads, apical contacts. Scale bar, 50 μm. (**H** and **I**) Percentage of EGFP^+^ cells either with apical contact in VZ (H), or in SVZ (I), of 44-day-old cCOs 2 dpe of EGFP plus either control, *NBPF14*, *NOTCH2NLB*, or *NOTCH2NLB* plus *NBPF14* expression plasmids. Data, mean of 9/10 control, 9/12 NOTCH2NLB, 12/12 NBPF14, and 10/12 NOTCH2NLB + NBPF14 electroporated organoids for (H)/(I), respectively; error bars, ±SD; ns not significant, *P < 0.05 and **P < 0.01 [Kruskal-Wallis test (H) and ANOVA with Dunnett’s correction (I)].

To explore a potential effect of *NBPF14* in the delamination of cNPCs, we first quantified the percentage of the RFP^+^ progeny of the microinjected APs in embryonic mouse neocortex that retained apical contact (i.e., contact to the ventricular surface) 24 and 48 hours after microinjection, i.e., that were aRG (Fig. 2, A to C). Notably, the NBPF14-progeny showed a reduction in the percentage of such cells 24 hours after microinjection (control, 66% versus NBPF14, 43%) (Fig. 2B).

This *NBPF14*-induced increase in losing apical contact was also observed 48 hours after microinjection, when the percentage of the RFP^+^ progeny without apical contact had increased for both the control and *NBPF14* condition, leaving only 13% of the NBPF14-progeny with apical contact in comparison to 29% in control (Fig. 2C). These data show that expression of *NBPF14* increases the first step of delamination of cNPCs from the VZ, that is, the loss of contact to the ventricular surface.

After losing their contact to the ventricular surface, cells destined to delaminate from the VZ migrate in the basal direction toward the SVZ and acquire BP identity (*6*, *8*, *11*). To examine this, we next quantified the distance from the ventricular surface of the cell body center of the progeny of microinjected APs in embryonic mouse neocortex at 24 and 48 hours after microinjection (Fig. 2, D to F). We found that at 24 hours, the cell body centers of the NBPF14-progeny were located on average 80 μm away from the ventricular surface, in comparison to 50 μm for the control, indicating that *NBPF14* caused a shift of a portion of the AP progeny toward the basal boundary of the VZ (Fig. 2E). Analysis of the positional distribution of the NBPF14-progeny (cell body centers) revealed that the two bins with the greatest percentages of RFP^+^ cells corresponded to the apical part of the SVZ (80 to 120 μm from the ventricular surface; control, 14% versus NBPF14, 34%) in embryonic mouse neocortex. In addition, compared to control, we detected a greater percentage of RFP^+^ cells located even further basally (>120 μm from the ventricular surface; control, 1% versus NBPF14, 15%) 24 hours after microinjection (fig. S3A).

At 48 hours after microinjection, the NBPF14-progeny was still found further away from the ventricular surface than in the control condition (average location of cell body centers: control, 76 μm versus NBPF14, 88 μm from the ventricular surface) (Fig. 2F). However, the distance from the ventricular surface increased only slightly from 24 to 48 hours after microinjection (24 hours, 80 μm versus 48 hours, 88 μm) (Fig. 2, E and F), suggesting that the first cell type in the lineage of delaminated cells originating from APs, i.e., the BPs, had reached their resident position in the SVZ. Furthermore, the positional distribution of the NBPF14-progeny showed that the two bins with the greatest percentages of RFP^+^ cells were located 80 to 120 μm away from the ventricular surface (80 to 120 μm; control, 21% versus NBPF14, 37%) (fig. S3B), a region comprising most of the SVZ in embryonic mouse neocortex at E14.5. Consistent with the concept of cNPC delamination from the VZ, the increase in basally located RFP^+^ cells upon *NBPF14* expression occurred at the expense of RFP^+^ cells located close to the ventricular surface (0 to 40 μm; control, 40% versus NBPF14, 26% at 24 hours; control, 25% versus NBPF14, 15% at 48 hours) (fig. S3, A and B). We conclude from these data with embryonic mouse neocortex that *NBPF14* increases the proportion of the AP progeny that undergoes delamination from the VZ, by increasing the proportion of AP progeny that loses contact to the ventricular surface and then migrates to the SVZ.

To corroborate these findings in chimpanzee cerebral organoids, we next determined the percentage of the EGFP^+^ progeny of transfected APs in the VZ that retained apical contact 2 dpe (Fig. 2, G and H). Consistent with our findings in embryonic mouse neocortex, we found a significant decrease in this percentage in *NBPF14*-electroporated organoids (Fig. 2H). We also determined the percentage of the EGFP^+^ progeny of transfected APs in the SVZ of the organoids 2 dpe. We detected an increase in this percentage in *NBPF14*-electroporated organoids compared to control-electroporated organoids (Fig. 2I), which corresponded to the *NBPF14*-induced decrease in the percentage of EGFP^+^ cells with apical contact (Fig. 2H). These data with chimpanzee cerebral organoids further support our conclusion that *NBPF14* leads to increased delamination of AP progeny from the VZ and their migration to the SVZ, consistent with these cells being newborn BPs.

### *NOTCH2NLB* increases the proportion of AP progeny that remains in the VZ in embryonic mouse neocortex and chimpanzee cerebral organoids

Upon microinjection of APs in embryonic mouse neocortex, *NOTCH2NLB* did not significantly affect the percentage of the progeny retaining apical contact, neither at 24 hours (Fig. 2B) nor at 48 hours (Fig. 2C). *NOTCH2NLB* also did not affect the average distance from the ventricular surface of the cell body centers of the AP progeny (Fig. 2E), or their distribution across the cortical wall (fig. S3A), 24 hours after microinjection. However, and in contrast to the NBPF14-progeny, 48 hours after microinjection, the NOTCH2NLB-progeny was on average significantly closer to the ventricular surface in comparison to control (control, 77 μm versus NOTCH2NLB, 50 μm) (Fig. 2F). Accordingly, analysis of the distribution of the NOTCH2NLB-progeny across the cortical wall revealed substantially more cells close to the ventricular surface compared to control (0 to 40 μm from ventricular surface; control, 25% versus NOTCH2NLB, 46%), and fewer cells in the basal regions (>100 μm from ventricular surface; control, 34% versus NOTCH2NLB, 14%) (fig. S3B).

These effects of *NOTCH2NLB* expression were even more notable when the analysis was confined to the portion of the AP progeny that retained apical contact 24 or 48 hours after microinjection. Thus, we detected a significantly shorter distance of the cell body centers to the ventricular surface in comparison to the control not only at 48 hours, but already at 24 hours (fig. S4, A and B). Analysis of their distribution across the VZ revealed a strong shift to the apical-most region at 24 hours and even more so at 48 hours (fig. S4, C and D). As this is the region where AP mitoses occur, these data suggest that the expression of *NOTCH2NLB* promotes mitosis of mouse APs, consistent with the data showing an increase in clone size (Fig. 1, B and C).

In contrast to AP microinjection in embryonic mouse neocortex (Fig. 2, B and C), chimpanzee cerebral organoids showed a significantly higher percentage of cells with apical contact in the VZ upon *NOTCH2NLB* electroporation (Fig. 2, G and H). Accordingly, we detected a small, but statistically significant decrease in the percentage of EGFP^+^ cells in the SVZ upon *NOTCH2NLB* electroporation (Fig. 2I). Together, our data demonstrate that *NOTCH2NLB* increases the percentage of AP progeny that remains in the VZ, retains apical contact, and undergoes mitosis in the apical-most region of the VZ, and hence increases the aRG pool size.

### *NBPF14* increases cNPC delamination from the VZ also in the presence of *NOTCH2NLB*

Expression of both *NOTCH2NLB* and *NBPF14* resulted in effects on the AP progeny that in essence were a combination of the individual effects of either *NOTCH2NLB* or *NBPF14* alone, as described in two previous sections. When the effects of *NOTCH2NLB* and *NBPF14* were opposite to each other, the combined expression of these two human-specific genes often yielded an intermediate outcome. Specific findings can be summarized as follows.

Upon microinjection of APs in embryonic mouse neocortex, *NBPF14* decreased the percentage of AP progeny that retained apical contact also in the presence of *NOTCH2NLB* (Fig. 2, B and C) and hence still induced the first step of cNPC delamination from the VZ. The effects of the combined expression of *NOTCH2NLB* and *NBPF14* on the average distance of the cell body centers from the ventricular surface yielded an intermediate result at 48 h, being similar to the control condition (Fig. 2, D to F). At this time point, the combined expression of *NOTCH2NLB* and *NBPF14* yielded a distribution of the AP progeny across the cortical wall that was essentially the same as that seen upon expression of *NBPF14* alone, and quite distinct from that seen upon expression of *NOTCH2NLB* alone. These data indicate that *NBPF14* also promotes the subsequent step of cNPC delamination from the VZ, that is, the migration of AP progeny to the SVZ (fig. S3B). This ability of *NBPF14* was confined to the portion of the AP progeny that had lost apical contact, as analysis of the distribution across the cortical wall of the AP progeny that retained apical contact revealed that here, the effect of *NOTCH2NLB* was dominant over that of *NBPF14*, with the pattern upon combined expression of *NOTCH2NLB* and *NBPF14* being essentially similar to that upon expression of *NOTCH2NLB* alone (fig. S4, C and D).

Upon expression in chimpanzee cerebral organoids, the coexpression of *NOTCH2NLB* and *NBPF14* yielded an intermediate outcome 2 dpe with regard to the percentage of the AP progeny in the VZ that retained apical contact, when compared to the expression of either *NOTCH2NLB* or *NBPF14* alone (Fig. 2, G and H). The same intermediate outcome 2 dpe was observed with regard to the percentage of the AP progeny that had migrated to the SVZ (Fig. 2I). Considering all these data together, we conclude that *NOTCH2NLB* and *NBPF14* affect cNPC activity and behavior in a synergistic manner, with *NOTCH2NLB* amplifying APs in the VZ and *NBPF14* promoting the delamination of AP progeny from the VZ.

### Enhanced delamination of AP progeny by *NBPF14* gives rise to increased bRG abundance

**Fig. 3.**
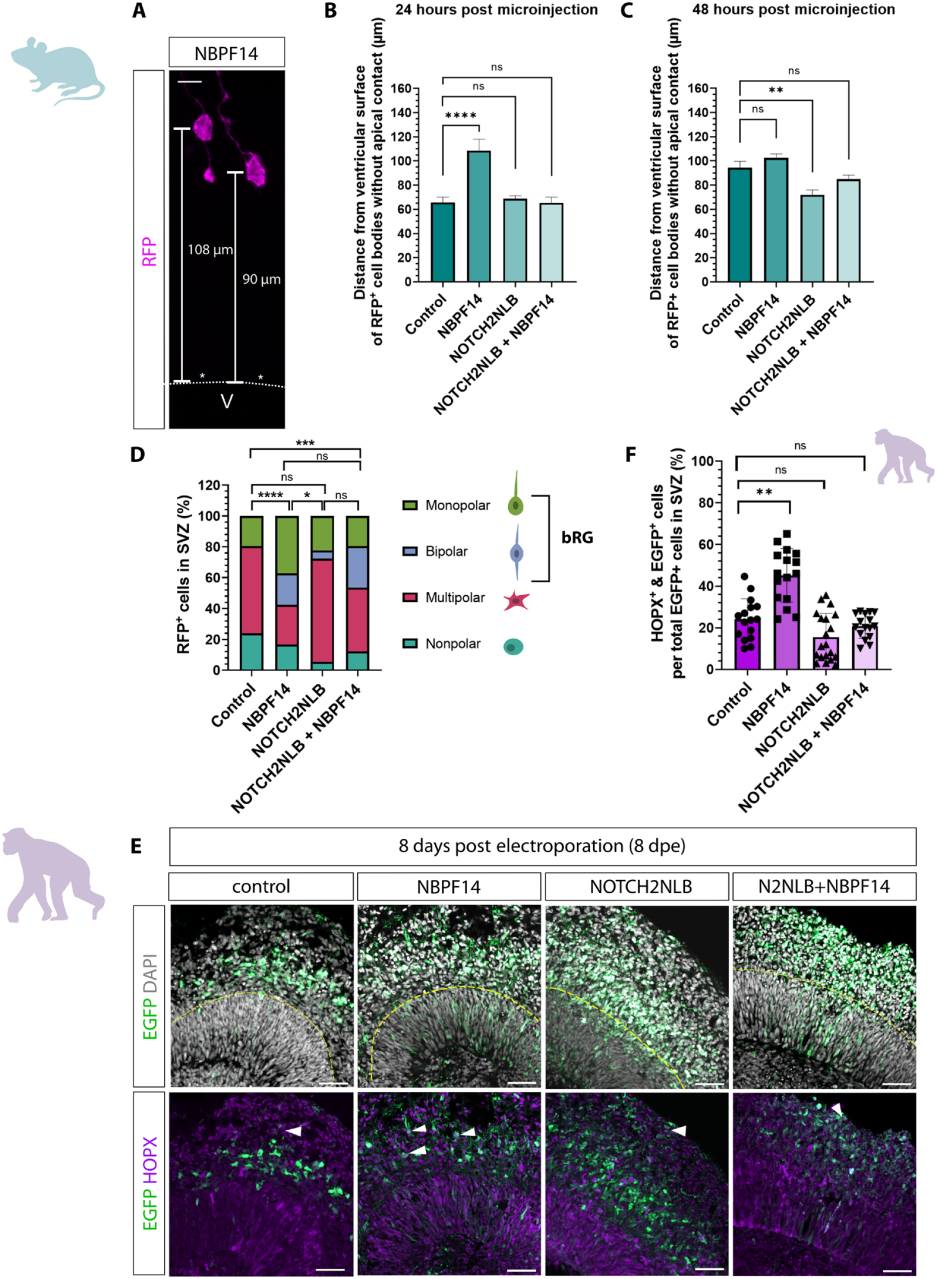
*NBPF14*-induced delamination of AP progeny results in increased bRG abundance. (**A**) RFP immunofluorescence 24 hours after automated microinjection (aMI) of *RFP* plus *NBPF14* mRNAs into single APs of E14.5 mouse neocortex (mNCX). Bars, distance of cell body center from ventricular surface (VS); asterisks, lack of apical contact; dashed line, VS; V, ventricle. Scale bar, 10 μm. (**B** and **C**) Distance of cell body center of RFP^+^ cells without apical contact from VS 24 (B) and 48 hours (C) after aMI of E14.5 mNCX of *RFP* (control) or *RFP* plus either *NBPF14*, *NOTCH2NLB*, or *NOTCH2NLB* plus *NBPF14* mRNAs. Data, mean of 38/96 control, 37/165 NBPF14, 62/87 NOTCH2NLB, and 62/165 NOTCH2NLB + NBPF14-progeny cells without apical contact of microinjected APs for (B)/(C), respectively. Data are a subset of the data in Fig. 2 (E and F); error bars, SEM; ns not significant, **P < 0.01 and ****P < 0.0001 (Kruskal-Wallis test). (**D**) Distribution of the morphologies (monopolar, green; bipolar, blue; multipolar, red; and nonpolar, turquoise) of RFP^+^ cells without apical contact in SVZ 48 hours after aMI of E14.5 mNCX of *RFP* (control) or *RFP* plus either *NBPF14*, *NOTCH2NLB*, or *NOTCH2NLB* plus *NBPF14* mRNAs. Data consist of 46 control, 78 NBPF14, 18 NOTCH2NLB, and 41 NOTCH2NLB + NBPF14-progeny cells without apical contact in SVZ; ns not significant, *P < 0.05, ***P < 0.001, and ****P < 0.0001 (Fisher’s exact test). (**E**) Double immunofluorescence for EGFP (green) and HOPX (purple), combined with DAPI (gray), of 50-day-old chimpanzee cerebral organoids (cCOs) 8 dpe of EGFP plus either control, *NBPF14*, *NOTCH2NLB*, or *NOTCH2NLB* plus *NBPF14* expression plasmids. Dashed lines, boundary VZ/SVZ; arrowheads, examples of EGFP/HOPX double-positive cells. Scale bars, 50 μm. (**F**) Percentage of EGFP^+^/HOPX^+^ cells in SVZ of 50-day-old cCOs 8 dpe. Data, mean of 16 control, 16 NBPF14, 20 NOTCH2NLB, and 16 NOTCH2NLB + NBPF14 electroporated organoids; error bars, ±SD; ns not significant, **P < 0.01 (Kruskal-Wallis test).

Further analysis of the delaminated portion of the AP progeny, i.e., the RFP^+^ cells without apical contact, after microinjection in embryonic mouse neocortex revealed three principal findings. First, the delaminated NBPF14-progeny had reached the SVZ at 24 hours and did not migrate further in the basal direction by 48 hours, indicating that the SVZ is their resident location, an observation consistent with these cells being BPs (Fig. 3, A to C, and fig. S5). Second, *NOTCH2NLB* expression seemed to delay the migration of a portion of the delaminated AP progeny to the SVZ (Fig. 3, A to C, and fig. S5). Third, combined expression of *NOTCH2NLB* plus *NBPF14* yielded an intermediate phenotype, with the distribution of the delaminated AP progeny across the cortical wall approaching that observed upon expression of *NBPF14* alone at 48 hours (Fig. 3, A to C, and fig. S5).

To distinguish between the different BP types, i.e., bIPs and bRG, we examined, after microinjection in embryonic mouse neocortex, the morphology of the delaminated cells that were located in the SVZ. bRG can be distinguished from bIPs by bearing radial processes, typically a basal process (*18*, *20*–*22*, *58*). Accordingly, we categorized the delaminated cells into four groups based on their morphology: monopolar (bRG), bipolar (bRG), multipolar (neurons and/or bIPs), and nonpolar (bIPs). In the control condition, as well as upon *NOTCH2NLB* expression, the overwhelming majority of the delaminated cells exhibited either multipolar or nonpolar morphology, with a minor percentage of the cells exhibiting monopolar or bipolar morphology (Fig. 3D). This reflects the typical distribution of BP types in embryonic mouse neocortex, in which bRG constitute a minority of BPs (*8*, *26*, *59*–*61*). In notable contrast, upon *NBPF14* expression, the majority of the delaminated cells in the SVZ exhibited either monopolar or bipolar morphology, indicating that these cells were bRG (Fig. 3D). Upon combined expression of *NOTCH2NLB* plus *NBPF14*, in comparison to expression of *NOTCH2NLB* alone, almost half of the BPs were now bRG, with an almost fivefold increase in the percentage of bipolar bRG (Fig. 3D). These data indicate that the expression of *NBPF14*, alone or in combination with *NOTCH2NLB*, increases the generation of bRG from APs in the embryonic mouse neocortex.

As bRG exhibit only low abundance in embryonic mouse neocortex, it was of interest to explore whether *NBPF14* would be able to increase bRG generation also in a species with high bRG abundance. For this purpose, we analyzed chimpanzee cerebral organoids 8 dpe, a time point at which EGFP^+^ progeny of electroporated APs can be detected in the SVZ (*54*). Specifically, we performed immunofluorescence for the radial glia marker homeodomain-only protein homeobox (HOPX) and restricted our analysis to the SVZ, where HOPX immunoreactivity is specific for bRG (Fig. 3, E and F) (*62*–*65*). Quantification of the percentage of EGFP^+^ cells in the SVZ that were positive for HOPX (i.e., bRG) showed that *NBPF14* expression increases this percentage nearly twofold. In contrast, we did not detect a significant change in this percentage upon *NOTCH2NLB* expression. Upon coexpression of *NOTCH2NLB* and *NBPF14*, this percentage remained similar to control (Fig. 3F).

Together, these data demonstrate that the enhanced delamination of AP progeny from the VZ upon *NBPF14* expression results in an increased abundance of a key cell type implicated in the evolutionary increase in cortical neurogenesis, and hence the expansion of the human neocortex (*6*, *20*, *21*, *26*, *27*), i.e., bRG, in embryonic mouse neocortex and chimpanzee cerebral organoids.

### *NBPF14* promotes nonvertical cleavage plane orientation of mitotic APs

**Fig. 4.**
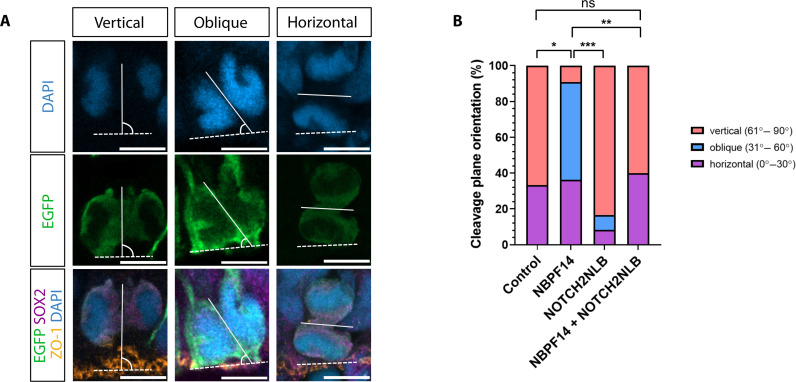
*NBPF14* promotes oblique at the expense of vertical cleavage plane orientation of mitotic APs. (**A**) Triple immunofluorescence for EGFP (green), ZO-1 (orange), and SOX2 (magenta), combined with DAPI staining (blue), of 44-day-old chimpanzee organoids 2 dpe with EGFP expression plasmid plus control plasmid (left) or *NBPF14* expression plasmid (NBPF14) (middle and right). Images show representative examples of mitotic APs in anaphase with vertical (left), oblique (middle), or horizontal (right) cleavage plane orientation. White lines indicate the axis of the cleavage plane deduced from the position of the sister chromatids and the concentration of SOX2 immunoreactivity near the future cleavage plane. Dashed white lines indicate the ventricular surface at the mitotic AP under study, deduced from the ZO-1 immunostaining. Scale bars, 5 μm. Cleavage plane angles are as follows 89° (vertical), 57° (oblique) and 3° (horizontal). (**B**) Distribution of the individual cleavage plane angles (categorized as vertical, 61° to 90°, light red; oblique, 31° to 60°, blue; and horizontal, 0° to 30°, violet) of EGFP-positive anaphase APs of 44-day-old chimpanzee cerebral organoids 2 dpe with EGFP expression plasmid plus either control plasmid (control), *NBPF14* expression plasmid (NBPF14), *NOTCH2NLB* expression plasmid (NOTCH2NLB), or *NOTCH2NLB* plus *NBPF14* expression plasmids (NOTCH2NLB + NBPF14). Data consists of nine control, 11 NBPF14, 12 NOTCH2NLB, and 10 NOTCH2NLB + NBPF14 anaphase APs of electroporated chimpanzee cerebral organoids; ns not significant, *P < 0.05, **P < 0.01, and ***P < 0.001 (Fisher’s exact test).

We sought to uncover the cell biological basis that underlies *NBPF14*’s ability to enhance the delamination of AP progeny from the VZ. To this end, we focused on cleavage plane orientation of mitotic APs as a major determinant of AP daughter cell delamination. While a vertical orientation of the cleavage plane, i.e., parallel to the apical-basal axis of APs, is known to be required for symmetric proliferative AP divisions (i.e., generating two APs), oblique or horizontal cleavage planes, the latter being oriented perpendicular to the apical-basal axis of APs, result in asymmetric differentiative or even symmetric consumptive AP divisions, leading to the delamination of one or even both AP daughter cells from the ventricular surface (*30*–*32*, *66*). To analyze a potential effect of *NBPF14* on cleavage plane orientation, we determined the cleavage plane orientation of mitotic APs in chimpanzee cerebral organoids 2 dpe. Specifically, we deduced the cleavage plane orientation of EGFP^+^ and SOX2^+^ mitotic APs from the position of the anaphase nuclei, and then determined the angle of this plane relative to the line of ventricular surface at the mitotic AP as marked by immunofluorescence for ZO-1 (referred to as cleavage plane angle; for details, see Materials and Methods) (Fig. 4A). We divided cleavage plane angles into three categories: (i) 61° to 90° as vertical cleavage, (ii) 31° to 60° as oblique cleavage, and (iii) 0° to 30° as horizontal cleavage. *NBPF14*-electroporated chimpanzee cerebral organoids in comparison to control showed a notable increase in oblique cleavage plane orientation of mitotic APs (oblique; control, 0% versus NBPF14, 55%) at the expense of vertical cleavage plane orientation (Fig. 4B). This strongly suggests that this change in cleavage plane orientation is an underlying cause for the increase in AP progeny delamination in the *NBPF14*-electroporated chimpanzee cerebral organoids (Fig. 2, B and C). In contrast, compared to *NBPF14*-electroporated organoids, *NOTCH2NLB*-electroporated organoids showed a notable increase in vertical cleavage plane orientation of mitotic APs (vertical; NBPF14, 9% versus NOTCH2NLB, 83%) at the expense of oblique and horizontal cleavage plane orientations (Fig. 4B). This in turn suggests that an increase in vertical cleavage plane orientation underlies the reduced AP progeny delamination and the increase in AP number in *NOTCH2NLB*-electroporated chimpanzee cerebral organoids. *NOTCH2NLB* + *NBPF14* co-electroporated chimpanzee cerebral organoids showed a distribution of cleavage plane orientations of mitotic APs that was similar to the control condition, suggesting that the effects of *NBPF14* and *NOTCH2NLB* on cleavage plane orientation canceled each other out (Fig. 4B).

### The combined expression of *NOTCH2NLB* and *NBPF14* increases BP abundance while maintaining the AP pool size

**Fig. 5.**
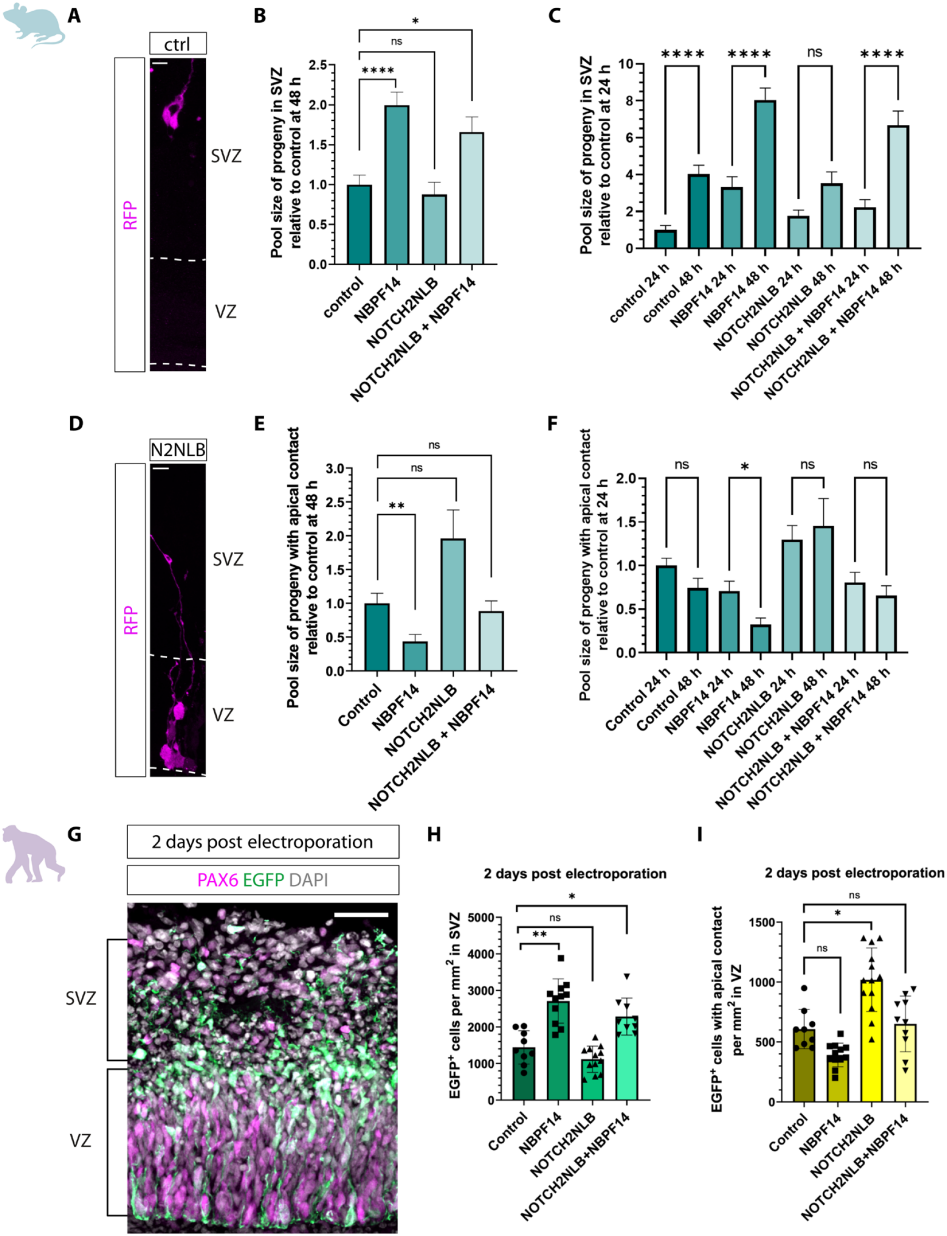
Combined expression of *NOTCH2NLB* and *NBPF14* increases BP abundance while maintaining the AP pool in embryonic mouse neocortex and chimpanzee cerebral organoids. (**A** and **D**) RFP immunofluorescence 48 hours after automated microinjection (aMI) of *RFP* mRNA [control, (A)] or *RFP* plus *NOTCH2NLB* [N2NLB, (D)] mRNAs into APs of E14.5 mouse neocortex (mNCX). Dashed lines, boundaries of VZ. Scale bars, 10 μm. (**B**, **C**, **E**, and **F**) Number of RFP^+^ cells either in SVZ [(B) and (C)], or with apical contact [(E) and (F)], relative to control 24 [(C) and (F)] or 48 hours [(B) and (E)] after aMI, 24 and 48 hours [(C) and (F)] or 48 hours [(B) and (E)] after aMI of E14.5 mNCX of *RFP* (control) or *RFP* plus either *NBPF14*, *NOTCH2NLB*, or *NOTCH2NLB* plus *NBPF14* mRNAs. Data for (B)/(E), mean of 61/63 control-, 72/75 NBPF14-, 41/43 NOTCH2NLB-, and 67/67 NOTCH2NLB + NBPF14–microinjected APs. Data [(C) and (F)], mean of 79/61 control-, 41/72 NBPF14-, 67/41 NOTCH2NLB-, and 58/67 NOTCH2NLB + NBPF14–microinjected APs 24/48 hours after aMI; error bars, SEM; ns not significant, *P < 0.05, **P < 0.01, and ****P < 0.0001 (Kruskal-Wallis test). Data are a subset of the data in Fig. 1C [(B) and (E)] and Fig. 1 (B and C) [(C) and (F)]. (**G**) EGFP (green) and PAX6 (magenta) immunofluorescence, combined with DAPI (gray), of a 44-day-old chimpanzee cerebral organoid (cCO) 2 dpe of EGFP plus control expression plasmids. Brackets, VZ and SVZ. Scale bar, 20 μm. (**H** and **I**) EGFP^+^ cells within 1 mm^2^ of SVZ area (H), or with apical contact in 1 mm^2^ of VZ area (I), of 44-day-old cCOs 2 dpe of EGFP plus either control, *NBPF14*, *NOTCH2NLB*, or *NOTCH2NLB* plus *NBPF14* expression plasmids. Data, mean of 9/9 control-, 11/12 NBPF14-, 12/13 NOTCH2NLB-, and 9/10 NOTCH2NLB + NBPF14–electroporated organoids for (H)/(I), respectively; error bars, ±SD; ns not significant, *P < 0.05 and **P < 0.01 (Kruskal-Wallis test).

After describing the various effects of *NBPF14*, *NOTCH2NLB*, and *NOTCH2NLB* + *NBPF14* on the behavior and properties of cNPCs, the question arises if these effects ultimately result in a change of the pool sizes of the different classes of cNPCs. To address this question, we first analyzed the pool size of the progeny without apical contact (i.e., BPs) in the SVZ 48 hours after microinjection into single APs of embryonic mouse neocortex (Fig. 5, A to C). We detected a similar increase in BP abundance for both *NBPF14* alone and in combination with *NOTCH2NLB* (NOTCH2NLB + NBPF14) (Fig. 5B). For the *NOTCH2NLB* condition, the pool size of the progeny without apical contact remained at the same level as the control (Fig. 5B). Moreover, by determining the development of the various BP pool sizes from 24 to 48 hours after microinjection, we found that BP abundance increased for the control, *NBPF14*, and *NOTCH2NLB* + *NBPF14* conditions, but not for the *NOTCH2NLB* condition, with the *NBPF14* and *NOTCH2NLB* + *NBPF14* conditions being at higher levels compared to the control condition (Fig. 5C). This indicates that the expression of *NBPF14* alone or in combination with *NOTCH2NLB* leads to a larger BP pool size in embryonic mouse neocortex, while expression of *NOTCH2NLB* alone does not.

Next, we analyzed the effects of the two human-specific genes on the AP pool size. For this purpose, we quantified the pool size of the AP progeny retaining apical contact (i.e., APs) 48 hours after microinjection into single APs of embryonic mouse neocortex (Fig. 5, D to F). *NOTCH2NLB* expression apparently doubled AP abundance (although due to the large variation in clone size in this condition, this increase was not statistically significant) (Fig. 5E). In contrast, expression of *NBPF14* resulted in a significant decrease in AP abundance (Fig. 5E). Upon coexpression of *NOTCH2NLB* and *NBPF14*, AP pool size was comparable to control (Fig. 5E). Moreover, by determining the development of the various AP pool sizes from 24 to 48 hours after microinjection, we found that AP abundance did not increase for the control, NOTCH2NLB, and NOTCH2NLB + NBPF14 conditions, but decreased for the NBPF14 condition (Fig. 5F). This indicates that the expression of *NBPF14* alone results in a reduction of the AP pool size, while the combined expression of *NOTCH2NLB* and *NBPF14* maintains the AP pool size at a similar level as the control.

To corroborate these findings, we quantified the number of EGFP^+^ cells in the SVZ of electroporated chimpanzee cerebral organoids 2 dpe to determine the effects of the two human-specific genes on the BP pool size in chimpanzee (Fig. 5, G and H). Consistent with our findings in embryonic mouse neocortex, we observed a significantly greater abundance of BPs in the SVZ upon *NBPF14* expression alone and in combination with *NOTCH2NLB*, but not upon *NOTCH2NLB* expression alone (Fig. 5H). Concerning the AP pool size in electroporated chimpanzee cerebral organoids, expression of *NOTCH2NLB* resulted in a significant increase in the pool size of EGFP^+^ cells retaining apical contact in the VZ compared to control-electroporated organoids (Fig. 5I). Similar to embryonic mouse neocortex, upon combined expression of *NOTCH2NLB* and *NBPF14*, AP abundance was comparable to that of the control (Fig. 5I).

In summary, expression of *NBPF14* alone results in an increase of the BP pool size, while reducing the AP pool size. Expression of *NOTCH2NLB* alone results in an increase of the AP pool size, while not affecting the BP pool size. The combined expression of *NOTCH2NLB* and *NBPF14* results in an increase in the BP pool size while maintaining the AP pool size.

## DISCUSSION

In the present study, we used a combination of automated microinjection into single APs of embryonic mouse neocortex and electroporation of APs in chimpanzee cerebral organoids to determine the effects of two human-specific genes, *NBPF14* and *NOTCH2NLB*, on the fate of the AP progeny. We find that *NBPF14* increases the delamination of AP progeny from the VZ, resulting in a greater abundance of bRG in the SVZ. In contrast, *NOTCH2NLB* increases the self-renewal of APs in the VZ, resulting in fewer AP progeny in the SVZ. These distinct effects of *NBPF14* and *NOTCH2NLB* are associated with opposing changes of these two human-specific genes on the cleavage plane orientation of mitotic APs. Whereas *NOTCH2NLB* increases vertical cleavages, which are required for increasing the AP pool size in the VZ, *NBPF14* increases oblique and horizontal cleavages, which are known to cause the delamination of AP progeny from the VZ (*32*, *66*, *67*). For *NOTCH2NLB*, our data provide further evidence corroborating the conclusion of two previous studies (*47*, *48*) that *NOTCH2NLB* promotes expansion of APs during neocortical development, and add insight into the mechanism underlying this process. By dissecting the effects of both *NBPF14* and *NOTCH2NLB*, our study offers a solution to the problem that a mere expansion of the AP pool size, without a concomitant increase in BP generation, as is the effect of *NOTCH2NLB*, would be insufficient for neocortex expansion. Specifically, we demonstrate that the pro–AP-progeny delamination effect of *NBPF14* constitutes the functional synergy with the pro-AP proliferation effect of *NOTCH2NLB* that is required for eventually increasing cortical neurogenesis and hence neocortex expansion. Moreover, our findings, summarized in Table 1, establish that the coevolution (*49*) and coexpression (*45*, *48*) of these two human-specific genes are of functional relevance.

**Table 1.** Summary of main findings. Arrows ↑, ↔, and ↓ refer to differences relative to control.

	Embryonic mouse neocortex	Chimpanzee cerebral organoid
NOTCH2NLB	Clone size (24 + 48 hours) ↑	Mitotic APs ↑, PCNA^+^ cells in VZ ↑
	Cells with apical contact (24 + 48 hours) ↔	Cells with apical contact ↑, vertical cleavage plane ↑
	Distance from ventricular surface, all cells ↔ (24 hours) ↓ (48 hours); distance from ventricular surface, cells without apical contact↔ (24 hours) ↓ (48 hours)	Proportion of AP progeny in SVZ ↓
	Cells with bRG morphology ↔	HOPX cells in SVZ ↔
	AP pool size ↔	AP pool size ↑
	BP pool size ↔	BP pool size ↔
NBPF14	Clone size (24 + 48 hours) ↔	Mitotic APs ↔, PCNA^+^ cells in VZ ↔
	Cells with apical contact (24 + 48 hours) ↓	Cells with apical contact ↓, Vertical cleavage plane ↓
	Distance from ventricular surface, all cells (24 + 48 hours) ↑; distance from ventricular surface, cells without apical contact ↑ (24 hours) ↔ (48 hours)	Proportion of AP progeny in SVZ ↑
	Cells with bRG morphology ↑	HOPX cells in SVZ ↑
	AP pool size ↓	AP pool size ↔
	BP pool size ↑	BP pool size ↑
NOTCH2NLB + NBPF14	Clone size ↑ (24 hours) ↔ (48 hours)	Mitotic APs ↑, PCNA^+^ cells in VZ ↔
	Cells with apical contact (24 + 48 hours) ↓	Cells with apical contact ↔, vertical cleavage plane ↔
	Distance from ventricular surface, all cells (24 + 48 hours) ↔; distance from ventricular surface, cells without apical contact (24 + 48 hours) ↔	Proportion of AP progeny in SVZ ↔
	Cells with bRG morphology ↑	HOPX cells in SVZ ↔
	AP pool size ↔	AP pool size ↔
	BP pool size ↑	BP pool size ↑

**Fig. 6.**
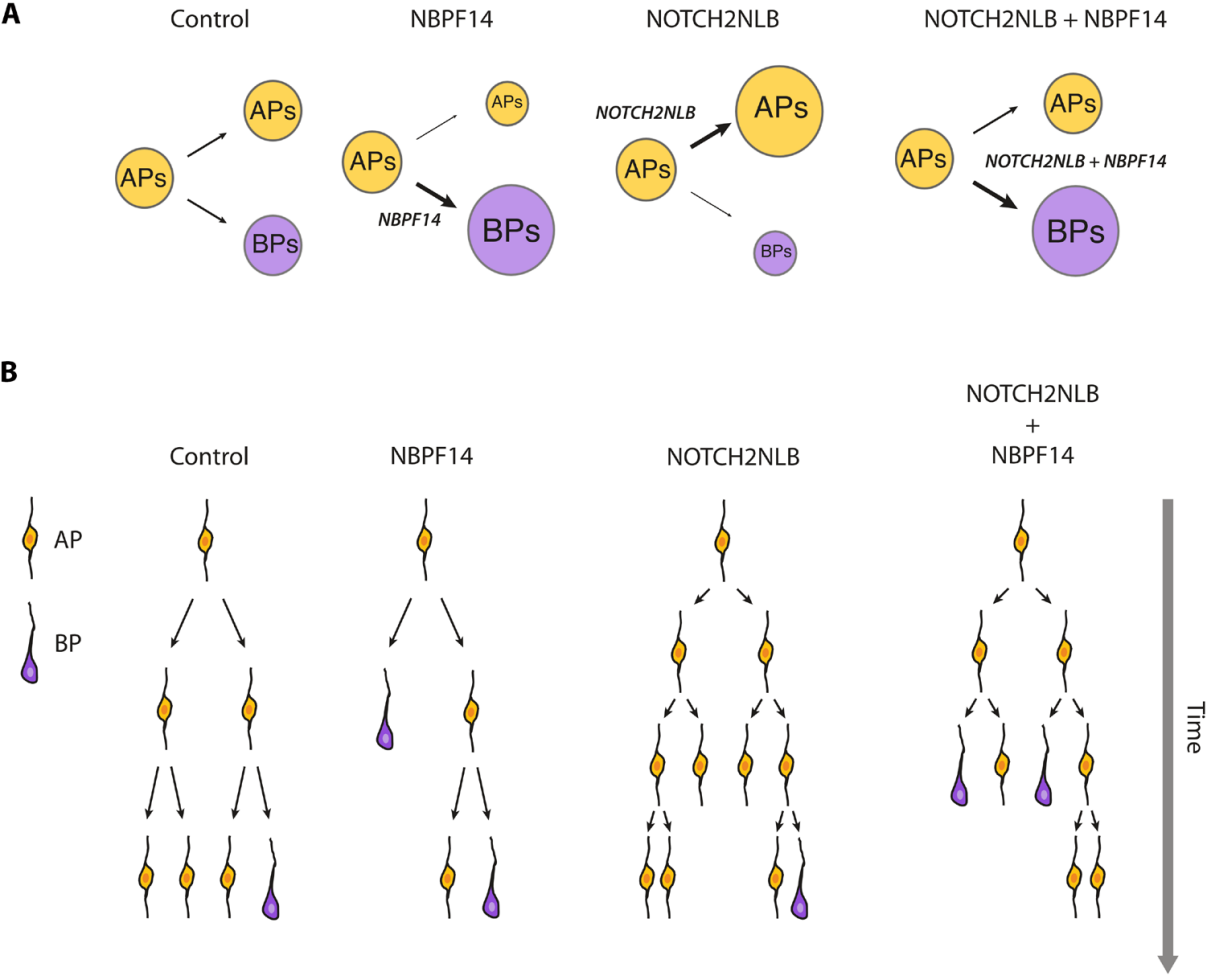
Effects of *NBPF14* and *NOTCH2NLB* on cNPCs. (**A**) Diagrams illustrating the effects of *NBPF14* (second diagram from left), *NOTCH2NLB* (third diagram from left), and *NOTCH2NLB* plus *NBPF14* (fourth diagram from left) on AP (yellow) and BP (purple) pool sizes in comparison to control (first diagram from left). (**B**) Diagrams illustrating the presumptive effects of *NBPF14* (second diagram from left), *NOTCH2NLB* (third diagram from left), and *NOTCH2NLB* plus *NBPF14* (fourth diagram from left) on the lineages from APs (yellow) to BPs (purple) in comparison to control (first diagram from left), to explain the changes in AP and BP pool sizes illustrated in (A). Note that fate and lineages of BPs are not illustrated.

Elaborating on the latter conclusion, our findings have the following implications for the role of *NBPF14* and *NOTCH2NLB* during cortical development (summarized in Fig. 6). First, if *NBPF14* had evolved without *NOTCH2NLB*, the expression of only *NBPF14* would lead to a relative reduction in the AP pool size over time due to the increased delamination of AP progeny. While in this scenario, there would initially be an increase in BP generation, in the long run, the abundance of BPs would be less than in the absence of *NBPF14* expression because the relative reduction in the AP pool size over time would diminish the source from which BPs are being generated (Fig. 6). This would ultimately result in a reduction in neuronal output, and hence in neocortex size. Second, if *NOTCH2NLB* had evolved without *NBPF14*, the expression of only *NOTCH2NLB* would lead to increased proliferation, i.e., increased self-renewal, of APs, resulting in a greater AP pool size. This, however, would imply that the generation of BPs from APs is reduced and/or delayed, resulting in a smaller BP pool size (Fig. 6). As BPs are the main source of cortical neurons, this in turn would imply a reduction in neuronal output, and hence in neocortex size. Third, the fact that *NBPF14* and *NOTCH2NLB* coevolved (*49*) and are coexpressed in cNPCs of developing neocortex (*45*) ensures that two effects of these two human-specific genes occur in parallel, (i) an increase in the AP pool size and (ii) an increased generation of BPs from APs, leading to a greater BP pool size. In other words, the combined expression of *NBPF14* and *NOTCH2NLB* results in an increased BP abundance while maintaining the AP pool (Fig. 6). This in turn gives rise to a higher neuronal output, and consequently to a larger neocortex.

In addition to the abovementioned implications for cortical development and evolution, our findings also suggest an impact on cortical malformations. *NOTCH2NLB* and *NBPF14* map to the 1q21.1 genomic region, which is associated with the 1q21.1 distal duplication/deletion syndrome, a neurodevelopmental disorder linked to various neurological symptoms including autism spectrum disorders, schizophrenia, microcephaly, and macrocephaly (*67*). The present functional data on *NBPF14* and its genomic localization (close to *NOTCH2NLB*) between the two breakpoints of the 1q21.1 distal duplication/deletion syndrome suggest *NBPF14* as a novel and likely important player in the macrocephaly/microcephaly associated with this syndrome. Moreover, a previous study showed premature neuronal differentiation in NOTCH2NLA and NOTCH2NLB KO brain organoids (*48*). In light of our data, this loss of *NOTCH2NLA* and especially of *NOTCH2NLB* would lead to an unbalanced effect of *NBPF14*, that is, increased delamination, consequently greater BP abundance, and eventually premature neuronal differentiation, in line with the report by Fiddes *et al.* (*48*)*.*

The two experimental approaches used in this study, automated microinjection into single APs of embryonic mouse neocortex and electroporation of APs in chimpanzee cerebral organoids, were complementary and yielded results that were largely in agreement to each other. Nevertheless, we noted three differences in the results obtained by these two approaches. The first two of these concern the effect of *NOTCH2NLB*, in the absence versus presence of *NBPF14* expression, with regard to the delamination of the AP progeny. Whereas in embryonic mouse neocortex, *NOTCH2NLB* expression did not affect this delamination, we observed a reduced delamination in chimpanzee cerebral organoids (see Table 1). Another difference with regard to delamination of the AP progeny could be detected upon coexpression of *NBPF14* and *NOTCH2NLB*. In this condition, coexpression of the two genes in embryonic mouse neocortex led to an increase in delamination (similar to the effect of *NBPF14*), whereas in chimpanzee cerebral organoids delamination was not changed (Table 1). Expression of *NBPF14* alone resulted in increased delamination in both embryonic mouse neocortex and chimpanzee cerebral organoids. In summary, in mouse neocortex, *NOTCH2NLB* expression does not affect or reduce delamination, whereas *NBPF14* expression increases it. Consequently, their coexpression leads to an overall increase in delamination. In chimpanzee cerebral organoids, expression of *NOTCH2NLB* reduces delamination, whereas expression of *NBPF14* increases it. Their coexpression results in balanced delamination, comparable to the control. This suggests that, in terms of delamination, APs in chimpanzee cerebral organoids respond more strongly to *NOTCH2NLB* expression than APs in embryonic mouse neocortex. These two different functional outcomes could be explained by differences in lineage commitment or responsiveness to NOTCH signaling between embryonic mouse neocortex and chimpanzee cerebral organoid development. As a third difference, whereas delamination was not reduced in embryonic mouse neocortex upon *NOTCH2NLB* microinjection, migration of the delaminated cells seemed to be delayed. This provides additional insight with relevance to the finding of Suzuki *et al.* (*47*) that *NOTCH2NLB* expression in embryonic mouse neocortex promotes the maintenance of cells in a progenitor state in the VZ. According to our data, these cells would already have delaminated and migrate, albeit with a slower pace, to the SVZ, suggesting that these cells are presumably newborn BPs.

To deepen our understanding of the functions of *NOTCH2NLB* and *NBPF14*, knockout and/or knockdown studies of these genes, either individually or in combination, would be helpful. However, because of the high sequence identity between either of these genes and their paralogs, it is extremely challenging to design specific and efficient CRISPR-Cas9 guide RNAs or small hairpin RNAs to generate knockouts or knockdowns that uniquely target either *NOTCH2NLB* or *NBPF14*. In this context, cerebral organoids generated from induced pluripotent stem cells (iPSCs) derived from patients with 1q21.1 distal deletion syndrome might provide valuable insights for future studies. Specifically, combining these organoids with rescue experiments involving *NOTCH2NLB* and *NBPF14*, either individually or together, using electroporation could be a fruitful approach to understand the role of these genes in the microcephaly associated with this syndrome. Another promising future direction would be to elucidate the molecular function of NBPF14. Here, one potential approach would be to identify NBPF14’s interaction partners by using a hemagglutinin (HA)–tagged version of this protein for pulldown assays, followed by mass spectrometry.

Recent studies on the functions of human-specific genes have revealed their critical roles in the expansion of the human neocortex. However, it is important to note that the size of the neocortex has progressively increased throughout mammalian evolution. This suggests that, beyond human- or species-specific genes, common and conserved mechanisms also underly cortical expansion in mammals (*68*). It has been proposed that increased activity in the extracellular signal–regulated kinase–bone morphogenetic protein 7–GLI family zinc finger 3 repressor (ERK–BMP7–GLI3R) signaling pathway plays a vital role in this evolutionary progression by extending the neurogenic period in the mammalian neocortex (*69*). Nevertheless, human-specific genes can also modulate these existing signaling pathways, as exemplified by *NOTCH2NLB* and its interaction with the NOTCH signaling pathway (*47*, *48*, *70*).

Previous studies concerning the functional role of human-specific genes during cortical development and evolution have analyzed only the function of single human-specific genes. Although these studies provided important insight [summarized in Tynianskaia and Heide (*70*)], corticogenesis is controlled by the activity of a variety of genes, including human-specific genes. To better understand cortical development and its evolution, the function of these genes needs to be investigated in a combinatory approach. Toward this goal, a recent study has reported the functional synergy of a human-specific and an ape-specific metabolic regulator in human neocortex development (*71*). To the best of our knowledge, the present study is the first one to perform a functional analysis of two human-specific genes and their interaction in cortical development. Specifically, we provide evidence that *NBPF14* and *NOTCH2NLB*, two human-specific genes that coevolved (*49*) and are coexpressed in cNPCs (*45*), act in a synergistic manner to ensure the correct balance of proliferation and lineage progression of cNPCs, which forms an essential basis of human neocortical expansion.

## MATERIALS AND METHODS

### Ethics

All animal experiments were conducted in accordance with the German Animal Welfare legislation (“Tierschutzgesetz”). All procedures regarding the animal experiments were approved by the regional Ethical Commission for Animal Experimentation of Dresden, Germany (Tierversuchskommission, Landesdirektion Dresden, license: 24-9168.24-9/2012-1). The human fetal material was provided by the Joint MRC/Wellcome Trust (grant no. MR/006237/1) Human Developmental Biology Resource (http://hdbr.org).

### Induced pluripotent stem cell lines

Chimpanzee Sandra A iPSC line (*72*, *73*) was cultured under feeder-free conditions on Matrigel (Corning)–coated dishes in mTeSR1 (Stemcell Technologies) with daily medium changes as previously described.

### Human fetal brain tissue

Human fetal (11 weeks postconception) brain tissue was dissected and shipped in Hibernate E media (GIBCO). Upon arrival, the tissue was fixed in 4% paraformaldehyde (PFA) in 120 mM phosphate buffer pH 7.4 for 3 hours at room temperature followed by 24 hours at 4°C. The specimen was then incubated overnight in phosphate-buffered saline (PBS) containing 30% sucrose at 4°C, embedded in Tissue-Tek OCT (Sakura) and frozen on dry ice. Cryosections of 20 μm thickness were cut on a cryostat (Microm HM 560, Thermo Fisher Scientific) and stored at −20°C until further use.

### Mice

Organotypic slices were prepared from E14.5 C57BL/6 mouse embryonic neocortex as previously described (*53*, *74*, *75*). In brief, the embryonic mouse neocortex was dissected at room temperature in Tyrode’s solution. After removal of the meninges, the tissue was embedded in 3% low-melting agarose (Agarose Wide Range, Sigma-Aldrich) in PBS at 37°C. After solidification of the agarose, coronal slices of 300 μm were dissected using a vibratome (Leica VT1000S, Leica). The slices were transferred to 37°C warm slice culture medium [SCM; neurobasal medium (Thermo Fisher Scientific), 10% rat serum (Charles River Japan), 2 mM l-glutamine (Thermo Fisher Scientific), 1× penicillin-streptomycin (Thermo Fisher Scientific), N_2_ supplement (Thermo Fisher Scientific), B27 supplement (Thermo Fisher Scientific), and 10 mM Hepes-NaOH, pH 7.3] and were stored in an incubator at 37°C in a humidified atmosphere of 40% O_2_/5% CO_2_/55% N_2_ until the start of microinjections.

### In vitro transcription of poly-A mRNA

For the microinjections, in vitro–transcribed poly-A mRNA was prepared as previously described (*53*, *74*, *75*). In brief, *NBPF14* and *NOTCH2NLB* genes were generated by gene synthesis and cloned into pBluescript II KS (+) by Genscript. For RFP, a previously described vector pTNT-RFP, was used for in vitro transcription (*74*). Vectors were linearized using restriction enzyme digestion and transcribed using the mMESSAGE mMACHINE T7 ULTRA transcription kit (Thermo Fisher Scientific) following the manufacturer’s instructions. Poly-A mRNA was dissolved in ribonuclease (RNase)–free bidistilled water at a concentration of 1 μg/μl, snap-frozen, and stored at −80°C until further use.

### Microinjection

Automated microinjection was performed as previously described (*53*, *74*, *75*). In brief, embryonic mouse neocortex slices were transferred to 37°C warm CO_2_-independent microinjection medium [DMEM-F12 (Sigma Aldrich) 2 mM l-glutamine (Thermo Fisher Scientific), 1× penicillin-streptomycin (Thermo Fisher Scientific), N_2_ supplement (Thermo Fisher Scientific), B27 supplement (Thermo Fisher Scientific), and 25 mM Hepes-NaOH, pH 7.3] and positioned on the microscope stage. Microinjection was performed with a microinjection mixture of Dextran Alexa 488 (Thermo Fisher Scientific) with RFP mRNA (250 ng/μl; control) or with RFP mRNA (250 ng/μl) together with either *NBPF14* mRNA (250 ng/μl), *NOTCH2NLB* mRNA (250 ng/μl), or *NBPF14* (250 ng/μl) and *NOTCH2NLB* mRNA (250 ng/μl) in RNase-free bidistilled water. Microinjection solution was loaded into micropipettes [pulled glass capillaries using a P-97 Flaming Brown micropipette puller (Sutter Instruments)] and automated microinjection was performed with the following settings: approach distance, 25 μm; depth, 10 μm; spacing, 15 μm; and speed, 100%.

### Slice culture and fixation of microinjected embryonic mouse neocortex slices

After microinjection slices were embedded in collagen and cultured in SCM in a whole-embryo culture incubator (Ikemoto Scientific Technology) at 37°C in a humidified atmosphere of 40% O_2_/5% CO_2_/55% N_2_ for the indicated times (24 or 48 hours). Afterward, slices were fixed in 4% PFA in 120 mM phosphate buffer pH 7.4 for 30 min at room temperature and for 24 hours at 4°C. The microinjected slices were then re-embedded into 3% low-melting agarose (Sigma-Aldrich). After solidification of the agarose, vibratome slices of 50 μm were cut parallel to the original cutting plane. Sections were collected floating in PBS in a 24-well plate. Floating sections were stored in PBS at 4°C until further use.

### Immunofluorescence staining of floating sections of microinjected embryonic mouse neocortex slices

Immunofluorescence staining of floating sections of microinjected embryonic mouse neocortex slices was performed as previously described (*53*, *74*, *75*). Antigen retrieval was performed in 0.01 M sodium citrate buffer (pH 6) containing 10% glycerol for 1 hour at 70°C. Primary antibodies were diluted in Tx buffer (0.2% gelatin, 300 mM NaCl, and 0.3% Triton X-100 in PBS), added to the slides, and incubated for at least 48 h. The following antibodies were used: RFP (rabbit polyclonal, 600-401-379, Rockland, 1:2000), RFP (mouse monoclonal, SAB2702202, Sigma-Aldrich, 1:1000), dextran (mouse monoclonal, 60026, Stemcell technologies, 1:1000), NOTCH2NL (mouse monoclonal, sc100307, Santa Cruz Biotechnology, 1:1000), and NBPF15 (pan-NBPF, rabbit polyclonal, HPA043105, Sigma-Aldrich, 1:1000). Secondary antibodies were 1:500 diluted in Tx buffer, added to the slides, and incubated for 1 h. The following secondary antibodies were used: anti-mouse Alexa 488 (donkey polyclonal, A-21202, Thermo Fisher Scientific), anti-mouse Alexa 555 (donkey polyclonal, A-31570, Thermo Fisher Scientific), anti-rabbit Alexa 488 (donkey polyclonal, A-21206, Thermo Fisher Scientific), and anti-rabbit Alexa 555 (donkey polyclonal, A-31572, Thermo Fisher Scientific). All floating sections were counterstained with 4′,6-diamidino-2-phenylindole (DAPI; 1:1000). Sections were mounted using Mowiol (Carl Roth).

### Generation of chimpanzee cerebral organoids

Chimpanzee iPSCs were differentiated into cerebral organoids as previously described (*54*, *55*, *72*, *73*, *76*, *77*). To generate embryoid bodies (EBs), 9000 cells per well were seeded in 96-well ultra-low attachment plates (Corning) in mTeSR1 containing 10 μM Y27632 (AbMole). Then cells were centrifuged for 3 min at 300*g*. After 48 hours, medium was changed to mTeSR1 without Y27632. On days 4 to 5 after seeding, medium was replaced with neural induction medium [DMEM/F12 (Thermo Fisher Scientific) containing 1% N_2_ supplement (Thermo Fisher Scientific), 1% GlutaMAX supplement (Thermo Fisher Scientific), 1% MEM nonessential amino acids (Thermo Fisher Scientific), and heparin (1 μg/ml; Sigma-Aldrich)] and changed every other day. On day 9 or 10 after seeding, embryoid bodies were embedded in Matrigel (Corning), and cultured in differentiation medium [1:1 DMEM/F12 (Thermo Fisher Scientific)/neurobasal (Thermo Fisher Scientific) containing 1% B27 supplement without vitamin A (Thermo Fisher Scientific), 0.5% N_2_ supplement (Thermo Fisher Scientific), 1% GlutaMAX supplement (Thermo Fisher Scientific), 0.5% MEM nonessential amino acids (Thermo Fisher Scientific), 1% penicillin-streptomycin (Thermo Fisher Scientific), 0.025% insulin solution (Sigma-Aldrich), and 0.00035% 2-mercaptoethanol (Merck)] on an orbital shaker with medium changes every other day. On day 15 or 16 after seeding, medium was switched to differentiation medium containing B27 supplement with vitamin A (Thermo Fisher Scientific). Cerebral organoids were further cultured in this differentiation medium until fixation, with medium changes every 2 to 3 days. All cerebral organoid culture steps were performed in an incubator at 37°C and in a humidified atmosphere of 5% CO_2_ and 95% air.

### Electroporation of chimpanzee cerebral organoids

For electroporation, *NBPF14* and *NOTCH2NLB* genes were cloned from pBluescript II KS (+) into pCaggs. Electroporation of cerebral organoids was performed as previously described (*54*, *55*). In brief, three to six ventricle-like structures per 42-day-old chimpanzee cerebral organoid were microinjected with the electroporation mixture containing expression plasmids (1500 ng/μl) according to table S1 combined with 0.1% Fast Green dye in PBS. The ventricle-like structures were microinjected with the mixture until they were visibly filled. Microinjected organoids were transferred to an in-house built petri dish electrode chamber connected to a square wave electroporator with a small amount of DMEM/F12. To ensure electroporation of the cells lining ventricle-like structures, cerebral organoids were placed in an orientation that the surfaces of the microinjected ventricle-like structures face toward the electrode connected to the positive pole of the electroporator. Subsequently, microinjected organoids were electroporated by applying five pulses of 80 V with a pulse duration of 50 ms and an interval of 1 s. After electroporation, cerebral organoids were transferred to a sterile 35-mm cell culture dish filled with prewarmed differentiation medium containing vitamin A and placed on an orbital shaker at 55 rpm in the incubator at 37°C in a humidified atmosphere of 5% CO_2_ and 95% air. Electroporated organoids were further cultured for 2 or 8 days in differentiation medium containing vitamin A with medium changes every 3 days before fixation for immunofluorescence analysis.

### Immunohistochemistry

Cerebral organoids were fixed at 2 or 8 dpe in 4% PFA in 120 mM phosphate buffer pH 7.4 for 30 min at room temperature. After incubation, cerebral organoids were washed three times in PBS and stored in PBS at 4°C until further use. For cryosectioning, fixed organoids were sequentially incubated in 15 and 30% sucrose in PBS overnight at 4°C. Cerebral organoids were embedded in cryomolds filled with TissueTek OCT (Sakura) and frozen on dry ice. Cryosections of 20 μm thickness were obtained using a Cryostat CryoStar NX70 (Thermo Fisher Scientific), captured on SuperFrost plus slides (Epredia), and stored at −20°C for later use. For immunofluorescence staining, human fetal brain and cerebral organoid sections were defrosted and washed in PBS (pH7.4). Antigen retrieval was performed in 0.1 M sodium citrate buffer (pH 6.0) for 1 hour at 70°C. After washing in PBS, sections were incubated in 0.3% Triton-X100/PBS, followed by incubation in 0.1 M glycine/PBS (pH 7.4). For blocking, sections were incubated with 15% fetal calf serum in PBS for 30 min. Primary antibodies were diluted 1:300 in Can Get Signal immunostain solution B (Toyobo) and incubated with sections at 4°C overnight. The following primary antibodies were used: GFP (chicken polyclonal, GFP-1020, Aves Lab), HOPX (rabbit polyclonal, PA5-90538, Thermo Fisher Scientific), NBPF15 (pan-NBPF, rabbit polyclonal, HPA043105, Sigma-Aldrich), NOTCH2NL (mouse monoclonal, sc100307, Santa Cruz Biotechnology), PAX6 (rabbit polyclonal, NBP1-89100, Novus Biologicals), PCNA (mouse monoclonal, CBL407, Merck), PH3 (rat monoclonal, ab10543, Abcam), SOX2 (goat polyclonal, AF2018, R&D systems), and ZO-1 (rabbit polyclonal, 61-7300, Thermo Fisher Scientific). The sections were washed in PBS and then incubated with secondary antibodies diluted 1:500 in Can Get Signal immunostain solution B and DAPI (1:1000) for 1 hour at room temperature. The following secondary antibodies were used: Alexa Fluor 488, chicken (goat polyclonal, A-11039, Thermo Fisher Scientific); Alexa Fluor 555, goat (donkey polyclonal, A-21432, Thermo Fisher Scientific), rabbit (donkey polyclonal, A-31572, Thermo Fisher Scientific), and mouse (donkey polyclonal, A-31570, Thermo Fisher Scientific); Alexa Fluor 647, goat (donkey polyclonal, A-21447, Thermo Fisher Scientific) and rabbit (donkey polyclonal, A-31573, Thermo Fisher Scientific). Sections were mounted using Mowiol (Carl Roth) and stored at 4°C in dark.

### Confocal microscopy

Confocal images were acquired with a Zeiss LSM 800 driven by ZEN Lite software and equipped with 10×, 20×, 40×, and 100× (oil) objectives and with a Zeiss LSM780 with a 63× Plan-Neofluar objective. For the microinjection experiments, images were taken as stacks of 0.6-μm optical sections. Cerebral organoid sections and human brain sections were imaged as stacks of 2- and 1-μm optical sections, respectively. When images were taken as tile scans, they were stitched together using the Zeiss ZEN software.

### Quantifications

All quantifications were done blindly. Images of microinjected embryonic mouse neocortex were analyzed using Fiji. Cell counts were performed in ZEN Lite or Fiji.

#### Clone size

The clone size of the AP progeny was determined by quantifying the number of RFP-positive cells, which were located less than 15 μm apart from each other in the horizontal direction.

#### Apical contact

The presence of apical contact was determined by tracing the apical process, if present, of a given RFP-positive cell toward the ventricular surface until it reached this surface or not. This analysis involved the checking of the stack of optical sections.

#### Distance to ventricular surface

The distance from the ventricular surface (defined as 0 μm) was measured with regard to the center of the cell body of a given RFP-positive cell following the radial organization of the cortical wall.

#### Distribution of AP progeny across the cortical wall

The distribution of the AP progeny across the cortical wall was determined by categorizing the distance to the ventricular surface of the RFP-positive cells into nine categories: 0 to 20 μm, >20 to 40 μm, >40 to 60 μm, >60 to 80 μm, >80 to 100 μm, >100 to 120 μm, >120 to 140 μm, >140 μm to 160 μm, and >160 μm.

#### Cerebral organoid quantifications

For quantification of electroporated chimpanzee cerebral organoids, organoids from at least three independent batches were used. For comparative analyses, ventricle-like structures exhibiting similar morphology with regard to the VZ (packing density of nuclei and radial organization), and SVZ, and a sufficiently high and roughly equal number of electroporated cells were quantified. The mean of several electroporated ventricle-like structures was calculated per organoid. The data obtained from the quantifications of electroporated samples are represented as a percentage of the EGFP-positive cell population or are expressed per indicated area of the EGFP-positive region.

#### VZ-SVZ boundary

The boundary between VZ and SVZ was defined on the basis of radial organization and density of nuclei.

#### Cleavage plane orientation

For the determination of the orientation of the cleavage plane, single EGFP-positive mitotic APs at the anaphase stage were analyzed. The cleavage plane was deduced from the position of the sister chromatids, which involved analysis of the stack of DAPI-stained optical sections (*78*, *79*), and the concentration of SOX2 immunoreactivity at the future cleavage plane. The angle of the cleavage plane was then determined relative to the ventricular surface, marked by ZO-1.

### Statistical analysis

Statistical analyses were performed using Prism (GraphPad Software). All data were analyzed for normal distribution using the Shapiro-Wilk test. Statistical significance was tested using either one-way analysis of variance (ANOVA) followed by Dunnett’s multiple comparison or Kruskal-Wallis test depending on the sample distribution or Fisher’s exact test for the analysis of contingency tables. Sample size for analysis of the progeny of microinjected cells and the number of electroporated cerebral organoids are indicated in the figure legends.
