# Supplementary material for: A dyad of human-specific NBPF14 and NOTCH2NLB orchestrates cortical progenitor abundance crucial for human neocortex expansion
Source: Sci Adv. 2025 Mar 26;11(13):eads7543. doi: 10.1126/sciadv.ads7543 (PMC11939065; doi:10.1126/sciadv.ads7543)
Supplement: Supplementary file 1 — Figs. S1 to S5 Table S1 [file sciadv.ads7543_sm.pdf]

Science Advances
AAAS

Supplementary Materials for

A dyad of human-specific *NBPF14* and *NOTCH2NLB* orchestrates cortical progenitor abundance crucial for human neocortex expansion

Nesil Eşiyok *et al.*

Corresponding author: Michael Heide, mheide@dpz.eu

Sci. Adv. **11**, eads7543 (2025)
DOI: 10.1126/sciadv.ads7543

This PDF file includes:

Figs. S1 to S5
Table S1

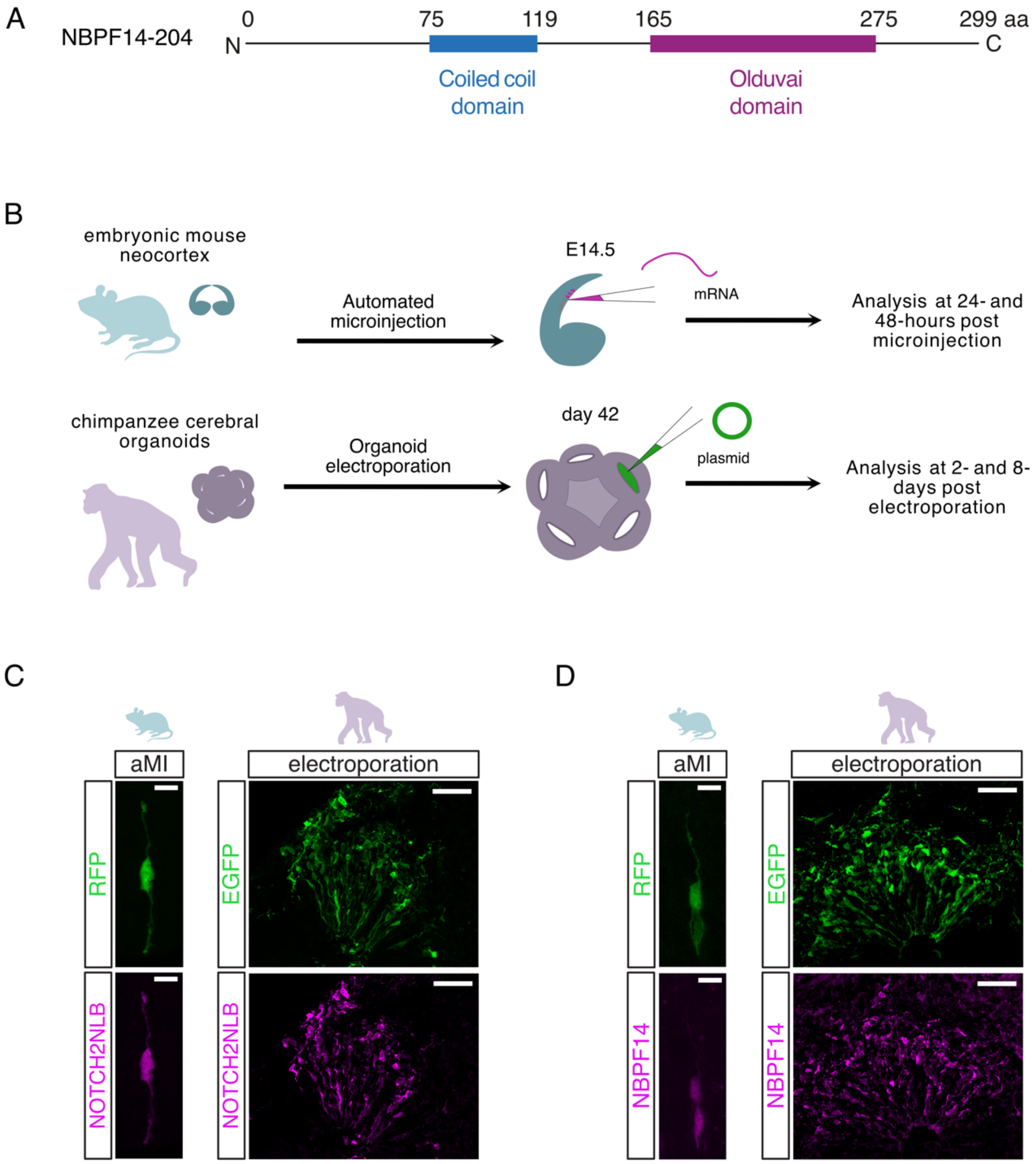

Fig. S1. Structure of the NBPF14 protein studied and experimental approaches used.
(A) Organization of protein domains of the NBPF14-204 isoform (ENST00000616120.4) subjected to functional investigation in this study. **(B)** Schematic representation of experimental approaches used to investigate the functions of *NBPF14* and *NOTCH2NLB* in embryonic mouse neocortex (mNCX) (upper part) and chimpanzee cerebral organoids (cCOs) (lower part) using automated microinjection and organoid electroporation, respectively. **(C)** Double immunofluorescence for NOTCH2NLB (magenta) and RFP (green, left) or EGFP (green, right) (i) of the progeny 24 h after automated microinjection (aMI) of *RFP* plus *NOTCH2NLB* mRNAs (left) into a single AP of an E14.5 embryonic mNCX and (ii) of a 44-days-old cCO 2 days after electroporation of EGFP expression plasmid plus *NOTCH2NLB* expression plasmid (right). Scale bars, 10 µm (left) and 50 µm (right). **(D)** Double immunofluorescence for NBPF (magenta) and

RFP (green, left) or EGFP (green, right) (i) of the progeny 24 h after automated microinjection (aMI) of *RFP* plus *NBPF14* mRNAs (left) into a single AP of an E14.5 embryonic mNCX and (ii) of a 44-days-old cCO 2 days after electroporation of EGFP expression plasmid plus *NBPF14* expression plasmid (right). Scale bars, 10 µm (left) and 50 µm (right).

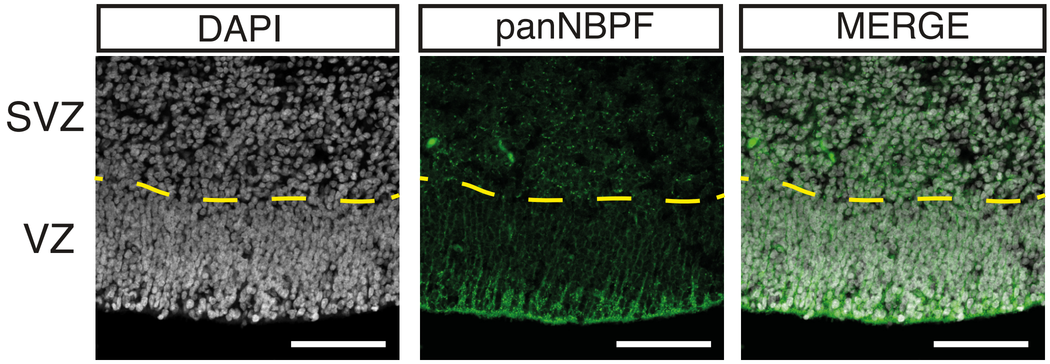

Fig. S2. Distribution of NBPF immunoreactivity in the VZ and SVZ of fetal human neocortex.

Immunofluorescence for NBPF (green), combined with DAPI staining (white), of fetal human neocortex tissue at 11 weeks post conception. Dashed yellow lines indicate the boundary between VZ and SVZ. Scale bars, 100 µm.

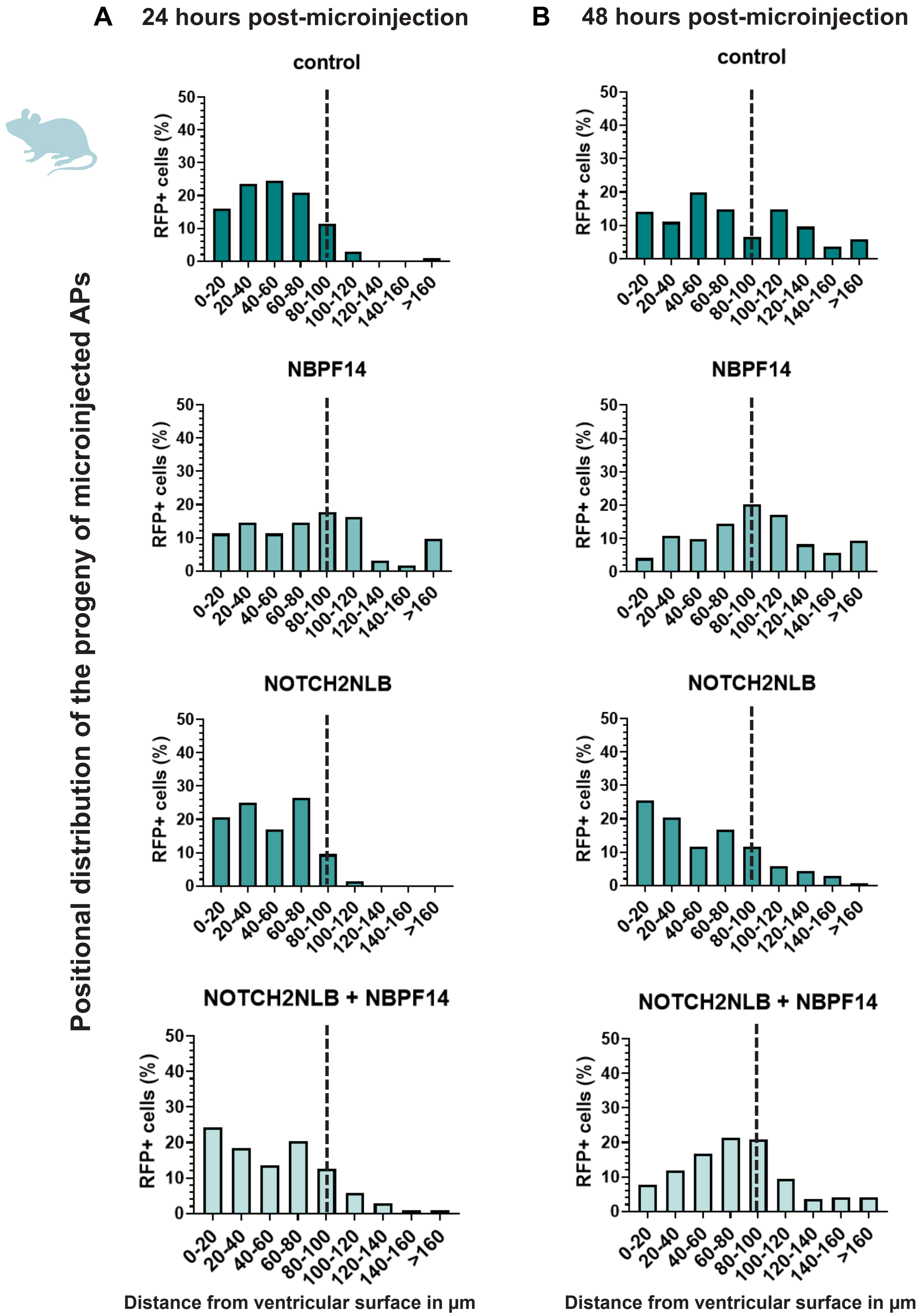
A
24 hours post-microinjection
B
48 hours post-microinjection
Positional distribution of the progeny of microinjected APs
control
NBPF14
NOTCH2NLB
NOTCH2NLB + NBPF14
RFP+ cells (%)
0
10
20
30
40
50
0-20
20-40
40-60
60-80
80-100
100-120
120-140
140-160
>160
Distance from ventricular surface in µm

Fig. S3. Distribution across the cortical wall of the RFP+ progeny of microinjected APs in embryonic mouse neocortex.

Distribution of the RFP+ cells across the cortical wall 24 h **(A)** and 48 h **(B)** after automated microinjection into single APs of E14.5 embryonic mouse neocortex of either *RFP* mRNA only (control), *RFP* plus *NBPF14* mRNAs (NBPF14), *RFP* plus *NOTCH2NLB* mRNAs (NOTCH2NLB), or *RFP* plus *NOTCH2NLB* plus *NBPF14* mRNAs (NOTCH2NLB + NBPF14). The distance of the cell body centers from the ventricular surface (0 µm) was determined, and cells were grouped into nine bins of 20 µm-steps each (0-20 µm, 20-40 µm, ..., >160 µm). For each of the four conditions at the two time points, the number of cells in each bin is expressed as a percentage of total (set to 100). Data consists of 106 control, 62 *NBPF14*, 136 *NOTCH2NLB* and 103 *NOTCH2NLB* plus *NBPF14* **(A)**, and of 136 control, 205 *NBPF14*, 137 *NOTCH2NLB* and 192 *NOTCH2NLB* plus *NBPF14* **(B)** progeny cells of microinjected APs. Dashed lines indicate the boundary between VZ and SVZ.

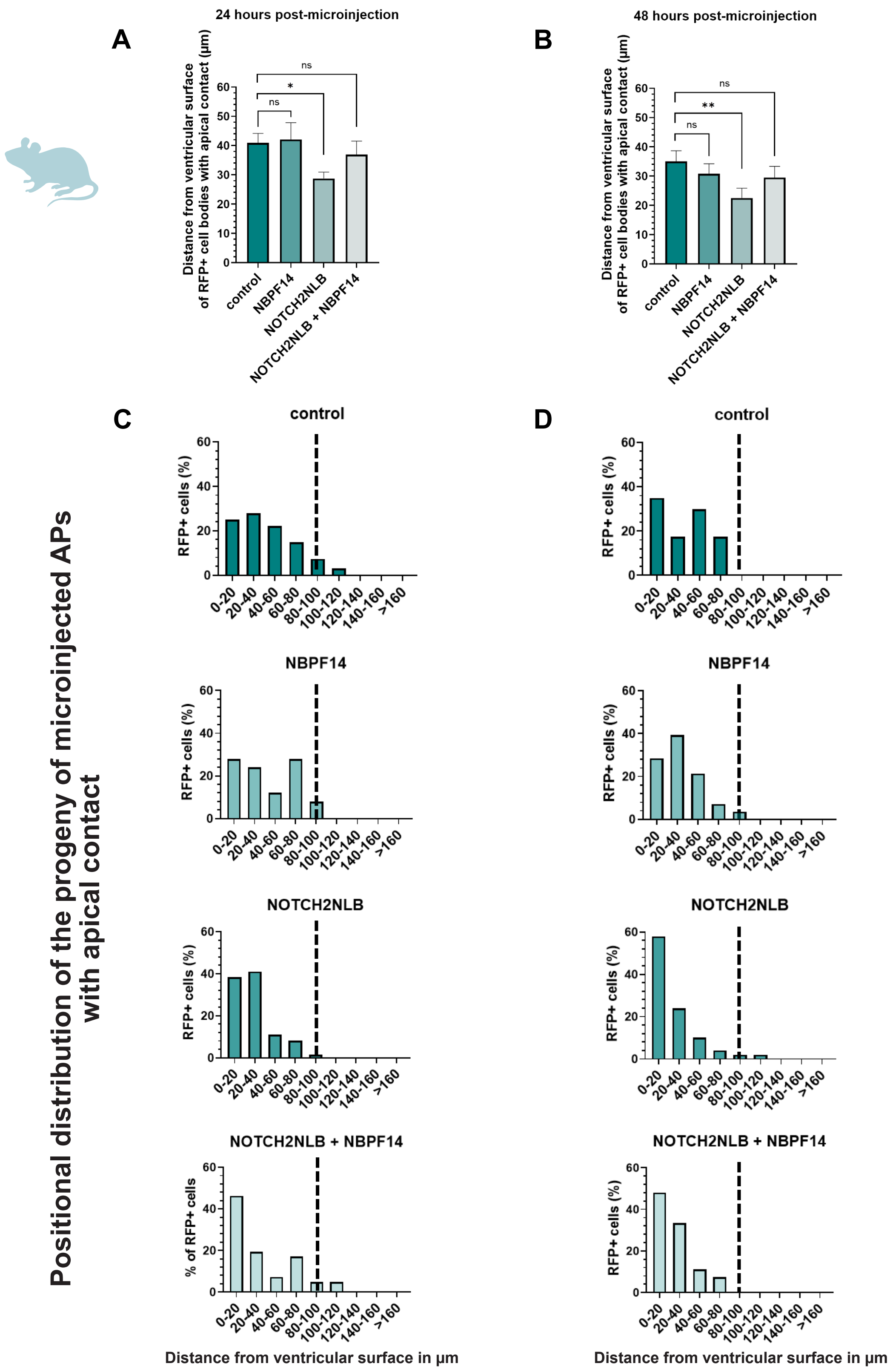

A
24 hours post-microinjection
Distance from ventricular surface of RFP+ cell bodies with apical contact (µm)
ns
*
ns
control
NBPF14
NOTCH2NLB
NOTCH2NLB + NBPF14
B
48 hours post-microinjection
Distance from ventricular surface of RFP+ cell bodies with apical contact (µm)
ns
**
ns
control
NBPF14
NOTCH2NLB
NOTCH2NLB + NBPF14
Positional distribution of the progeny of microinjected APs with apical contact
C
D
control
NBPF14
NOTCH2NLB
NOTCH2NLB + NBPF14
RFP+ cells (%)
% of RFP+ cells
0-20
20-40
40-60
60-80
80-100
100-120
120-140
140-160
>160
Distance from ventricular surface in µm

Fig. S4. Distance from the ventricular surface of the apical contact-retaining RFP+ progeny of microinjected APs and their distribution across the cortical wall in embryonic mouse neocortex.

(A-B) Quantification of the distance from the ventricular surface of the cell body center of RFP+ cells retaining apical contact 24 h **(A)** and 48 h **(B)** after automated microinjection into single APs of E14.5 embryonic mouse neocortex (mNCX) with either *RFP* mRNA only (control), *RFP* plus *NOTCH2NLB* mRNAs (NOTCH2NLB), *RFP* plus *NBPF14* mRNAs (NBPF14), or *RFP* plus *NOTCH2NLB* plus *NBPF14* mRNAs (NOTCH2NLB + NBPF14). Data are the mean of 68 control, 74 *NOTCH2NLB*, 25 *NBPF14* and 41 *NOTCH2NLB* plus *NBPF14* **(A)**, and of 40 control, 50 *NOTCH2NLB*, 40 *NBPF14* and 27 *NOTCH2NLB* plus *NBPF14* **(B)** progeny cells retaining apical contact of microinjected APs. Note that this data set is a subset of the data shown in Figure 2E-F; error bars indicate SEM; ns not significant, *P <0.05, **P <0.01 (Kruskal-Wallis test). **(C-D)** Distribution of the RFP+ cells that retain apical contact across the cortical wall 24 h **(C)** and 48 h **(D)** after automated microinjection into single APs of E14.5 embryonic mNCX of either *RFP* mRNA only (control), *RFP* plus *NBPF14* mRNAs (NBPF14), *RFP* plus *NOTCH2NLB* mRNAs (NOTCH2NLB), or *RFP* plus *NOTCH2NLB* plus *NBPF14* mRNAs (NOTCH2NLB + NBPF14). The distance of the cell body centers from the ventricular surface (0 μm) was determined, and cells were grouped into nine bins of 20 μm-steps each (0-20 μm, 20-40 μm, ..., >160 μm). For each of the four conditions at the two time points, the number of cells in each bin is expressed as a percentage of total (set to 100). Data consists of 68 control, 25 *NBPF14*, 74 *NOTCH2NLB* and 41 *NOTCH2NLB* plus *NBPF14* **(C)**, and of 40 control, 40 *NBPF14*, 50 *NOTCH2NLB* and 27 *NOTCH2NLB* plus *NBPF14* **(D)** progeny cells retaining apical contact of microinjected APs. Note that this data set is a subset of the data shown in Figure S3. Dashed lines indicate the boundary between VZ and SVZ.

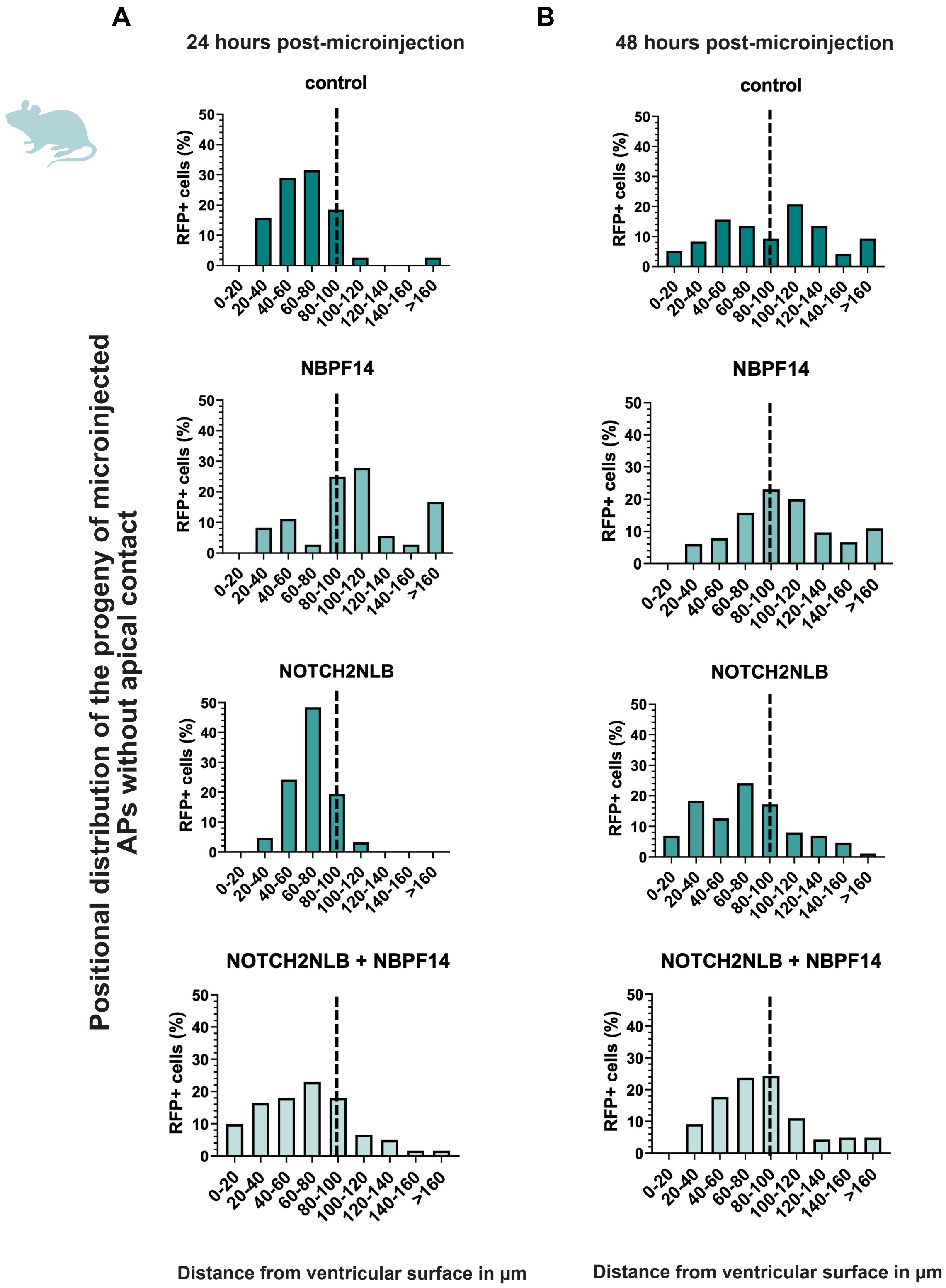

Fig. S5. Distribution across the cortical wall of the apical contact-lacking RFP+ progeny of microinjected APs in embryonic mouse neocortex.

Distribution of the RFP+ cells that lack apical contact across the cortical wall 24 h **(A)** and 48 h **(B)** after automated microinjection into single APs of E14.5 embryonic mouse neocortex of either *RFP* mRNA only (control), *RFP* plus *NBPF14* mRNAs (NBPF14), *RFP* plus *NOTCH2NLB* mRNAs (NOTCH2NLB), or *RFP* plus *NOTCH2NLB* plus *NBPF14* mRNAs (NOTCH2NLB + NBPF14). The distance of the cell body centers from the ventricular surface (0 µm) was determined, and cells were grouped into nine bins of 20 µm-steps each (0-20 µm, 20-40 µm, ..., >160 µm). For each of the four conditions at the two time points, the number of cells in each bin is expressed as a percentage of total (set to 100). Data consists of 38 control, 37 *NBPF14*, 62 *NOTCH2NLB* and 62 *NOTCH2NLB* plus *NBPF14* **(A)**, and of 96 control, 165 *NBPF14*, 87 *NOTCH2NLB* and 165 *NOTCH2NLB* plus *NBPF14* **(B)** progeny cells lacking apical contact of microinjected APs. Note that this data set is a subset of the data shown in Figure S3. Dashed lines indicate the boundary between VZ and SVZ.

Table S1. Composition of the electroporation mix.

	**Control**	**NBPF14**	**NOTCH2NLB**	**NBPF14 + NOTCH2NLB**
pCAGGS-EGFP	500 ng/µl	500 ng/µl	500 ng/µl	500 ng/µl
pCAGGS (empty)	1000 ng/µl	500 ng/µl	500 ng/µl	–
pCAGGS-NBPF14	–	500 ng/µl	–	500 ng/µl
pCAGGS-NOTCH2NLB	–	–	500 ng/µl	500 ng/µl
Total plasmid	1500 ng/µl	1500 ng/µl	1500 ng/µl	1500 ng/µl
